# *MGMT *promoter methylation in gliomas-assessment by pyrosequencing and quantitative methylation-specific PCR

**DOI:** 10.1186/1479-5876-10-36

**Published:** 2012-03-06

**Authors:** Annette Bentsen Håvik, Petter Brandal, Hilde Honne, Hanne-Sofie Spenning Dahlback, David Scheie, Merete Hektoen, Torstein Ragnar Meling, Eirik Helseth, Sverre Heim, Ragnhild A Lothe, Guro Elisabeth Lind

**Affiliations:** 1 Section for Cancer Cytogenetics, Institute for Medical Informatics, Oslo University Hospital-The Norwegian Radium Hospital, P.O. Box 4950 Nydalen, N-0424 Oslo, Norway; 2 Department of Cancer Prevention, Institute for Cancer Research, Oslo University Hospital-The Norwegian Radium Hospital, Oslo, Norway; 3 Centre for Cancer Biomedicine, Faculty of Medicine, University of Oslo, Oslo, Norway; 4 Faculty of Medicine, University of Oslo, Oslo, Norway; 5 Department of Oncology, Oslo University Hospital-The Norwegian Radium Hospital, Oslo, Norway; 6 Department of Pathology, Oslo University Hospital-Rikshospitalet, Oslo, Norway; 7 Department of Neurosurgery, Oslo University Hospital-Rikshospitalet, Oslo, Norway; 8 Department of Neurosurgery, Oslo University Hospital-Ullevål Hospital, Oslo, Norway; 9 Cancer stem cell innovation center, Oslo University Hospital, Oslo, Norway

**Keywords:** Glioma, Glioblastoma, *MGMT*, Methylation, Gene expression, Low-grade glioma, High-grade gliomas, Pyrosequencing, qMSP, RT-PCR

## Abstract

**Background:**

Methylation of the O^6^-methylguanine-DNA methyltransferase (*MGMT*) gene promoter is a favorable prognostic factor in glioblastoma patients. However, reported methylation frequencies vary significantly partly due to lack of consensus in the choice of analytical method.

**Method:**

We examined 35 low- and 99 high-grade gliomas using quantitative methylation specific PCR (qMSP) and pyrosequencing. Gene expression level of MGMT was analyzed by RT-PCR.

**Results:**

When examined by qMSP, 26% of low-grade and 37% of high-grade gliomas were found to be methylated, whereas 97% of low-grade and 55% of high-grade gliomas were found methylated by pyrosequencing. The average *MGMT *gene expression level was significantly lower in the group of patients with a methylated promoter independent of method used for methylation detection. Primary glioblastoma patients with a methylated *MGMT *promoter (as evaluated by both methylation detection methods) had approximately 5 months longer median survival compared to patients with an unmethylated promoter (log-rank test; pyrosequencing *P *= .02, qMSP *P *= .06). One third of the analyzed samples had conflicting methylation results when comparing the data from the qMSP and pyrosequencing. The overall survival analysis shows that these patients have an intermediate prognosis between the groups with concordant *MGMT *promoter methylation results when comparing the two methods.

**Conclusion:**

In our opinion, *MGMT *promoter methylation analysis gives sufficient prognostic information to merit its inclusion in the standard management of patients with high-grade gliomas, and in this study pyrosequencing came across as the better analytical method.

## Background

Gliomas are histologically divided into several subgroups including astrocytomas, oligodendrogliomas, and oligoastrocytomas and are graded from I to IV according to the WHO classification [1]. Prognosis is highly variable depending on histopathology, grade, patient age, and genetic tumor factors such as the presence of a 1p/19q co-deletion, *IDH1 *and *IDH2 *mutations, and *MGMT *promoter methylation [1,2]. The most common glioma subtype in adults is glioblastoma (GBM) with an annual incidence of 3-4/100 000 [1]. This is also the subgroup with the least favorable prognosis. In 2005, Stupp and coworkers reported a 2.5 months increase in median overall survival for GBM patients when adding concomitant and adjuvant temozolomide (TMZ) to postoperative radiotherapy [3]. It should be noted, however, that clinical trials tend to report higher median overall survival rates than retrospective studies, possibly due to selection bias [1]. Therefore, it is not surprising that a retrospective population-based Norwegian study reported a lower median overall survival for GBM patients (9.9 months) than that of the Stupp study patients (14.6 months and 12.1 months) [3,4].

About 5% of the DNA methylation induced by TMZ is located at the O^6^-position of guanine and methylation in this position is considered to be the main contributor to the cytotoxic effect [5-7]. The DNA repair enzyme O-6-methylguanine-DNA methyltransferase (MGMT) removes methyl groups from the O^6^-position of guanine and the expression of MGMT is therefore thought to inhibit the cytotoxic effect of TMZ [5,6]. Even though the first studies suggesting that *MGMT *promoter hypermethylation was an important molecular marker in high-grade gliomas were published almost a decade ago [8-10], the extent of its positive prognostic and predictive value in the different grades of gliomas remains to be determined [11]. Further, though several studies indicate that *MGMT *promoter methylation is a prognostic marker [11], there is no clear consensus as to which detection method should be preferred or what constitutes optimal threshold values for scoring samples as methylation positive. As a result, a wide range of reported glioma *MGMT *methylation frequencies can be seen (Additional file 1: Tables S1 and S2) [11]. We have used two independent quantitative methylation detection methods, quantitative methylation specific polymerase chain reaction (qMSP) and pyrosequencing, to analyze a large series of gliomas. We also analyzed the gene expression level of *MGMT *in the majority of these samples. To illustrate the variability in methylation frequencies and methylation detection methods, we systemized publications reporting *MGMT *promoter methylation in a tabular overview (Additional file 1: Tables S1 and S2).

## Materials and methods

### Patients and samples

Tumor samples from 134 glioma patients (diffuse astrocytoma WHO grade II (n = 10), oligodendroglioma WHO grade II (n = 6), oligoastrocytoma WHO grade II (n = 17), low-grade neuroepithelial tumour not otherwise specified (n = 2), anaplastic astrocytoma WHO grade III (n = 4), anaplastic oligodendroglioma WHO grade III (n = 6), anaplastic oligoastrocytoma WHO grade III (n = 3), glioblastoma WHO grade IV (n = 86)) and four meningioma patients who underwent surgery at the Department of Neurosurgery (Oslo University Hospital) between January 2005 and January 2009 were included in this study. The meningioma samples served as *MGMT *promoter methylation negative controls [12]. Histological diagnoses were reviewed by an expert neuropathologist (author D.S.). Patients alive were included following written, informed consent whereas permission to include deceased patients was obtained from The National Health Authorities. The study was approved by the Regional Ethics Committee (S-06046) as well as the Institutional Study Board.

### DNA isolation and bisulfite conversion

DNA was extracted from fresh frozen tissue using a standard phenol-chloroform procedure and its quantity and quality was measured using a NanoDrop ND-1000 Spectrophotometer (Thermo Fisher Scientific). Unmethylated cytosine residues were converted to uracil by bisulfite treatment of 1.3 μg DNA using the EpiTect Bisulfite Kit (Qiagen) according to the manufacturers' protocol. After conversion, DNA was eluted in buffer (Qiagen) to a final concentration of 30 ng/μl.

### Quantitative methylation specific polymerase chain reaction

*MGMT *promoter methylation was quantitatively assessed by two qMSP assays, each covering 11 CpG sites (CpGs). The two assays analyzed CpGs in partially overlapping regions (Additional file 1: Figure S1), but detected methylation on opposite DNA strands. Primers (Medprobe) and 6-FAM labeled minor groove binder (MGB) probes (Applied Biosystems, Life Technologies) were modified from two previously reported assays [13,14] to adjust the melting temperature to 60°C for primers and 70°C for probes. Amplification of a part of the *ALU*-element (ALU C4) was used for normalization [15]. Primers and probe sequences are listed in Table 1. Amplification reactions were carried out in triplicate in 384 well plates using the 7900HT Fast-Real time PCR machine (Applied Biosystems, Life Technologies). The total reaction volume was 20 μl and contained 30 ng bisulfite treated DNA, 0.9 μM forward and reverse primer, 0.2 μM probe, and 1× TaqMan Universal PCR Mastermix (No AmpErase UNG; Applied Biosystems, Life Technologies). The PCR program included initial denaturation at 95°C for 10 min followed by 45 cycles of 95°C for 15 s and 60°C for 60 s. Results were processed and exported using default settings in the software SDS 2.2.2 (Applied Biosystems, Life Technologies). Each plate included several non-template controls (water), an unmethylated control (bisulfite treated normal leukocyte DNA), and a methylated control (bisulfite converted in vitro methylated human DNA; Chemicon, Millipore). To quantitate the amount of fully methylated alleles in each reaction, a standard curve was generated for each plate using a serial dilution of the methylated control (32.5-0.052 ng).

**Table 1:** Primers and probes used for quantitative methylation-specific polymerase chain reaction (qMSP)

Assay	Sequence
**MGMT qMSP**^**a**^	

Forward primer	GCGTTTCGACGTTCGTAGGT

Reverse primer	CACTCTTCCGAAAACGAAACG

Probe	6FAM-AAACGATACGCACCGCGA-MGB

**MGMT_1 qMSP**^**b**^	

Forward primer	CGAATATACTAAAACAACCCGCG

Reverse primer	TTTTTTCGGGAGCGAGGC

Probe	6FAM-CGCGATACGCACCGTTTACG-MGB

**ALU qMSP**^**c**^	

Forward primer	GGTTAGGTATAGTGGTTTATATTTGTAATTTTAGTA

Reverse primer	ATTAACTAAACTAATCTTAAACTCCTAACCTCA

Probe	6FAM-CCTACCTTAACCTCCC-MGB

*Abbreviations: qMSP*, quantitative methylation-specific polymerase chain reaction; *MGB*, minor groove binder

a) Modified after Rivera et al., Neuro Oncol. (2010)

b) Modified after Hoque et al., J. Natl. Cancer Inst. (2006)

c) Weisenberger et al., Nucleic Acids Res. (2005)

Samples with a Ct-value above 35 were censored (resulting in a quantity of 0). The percentage of methylated reference (PMR) was calculated for each sample from the median quantity value from the triplicates by dividing the MGMT/ALU quantity ratio in the target by the MGMT/ALU quantity ratio in the methylated control, and multiplying by 100. A threshold value for scoring methylation positive samples was defined based on the qMSP result of four meningiomas, which all had PMR values of zero in both qMSP assays. Only samples with a PMR value above zero in both assays were scored as methylation positive. Representative PCR products from both reactions were sequenced in order to verify the fragment identity.

### Pyrosequencing

Five CpG sites in the *MGMT *promoter were analyzed by pyrosequencing using the PyroMark MD System (Qiagen). Bisulfite treated DNA was amplified in a PCR reaction using primers from the PyroMark Q96 CpG MGMT kit (part number 972032, Qiagen). In addition to the samples, each run included a non-template control (water), an unmethylated control (bisulfite treated normal leukocyte DNA), and a methylated control (bisulfite converted in vitro methylated human DNA). The amplification was carried out in 96-well plates and the PCR reaction and cycling conditions were according to the kit manual. Subsequent sample preparation and pyrosequencing was performed as described in the PyroMark MD Sample Prep Guidelines. In brief, the double stranded PCR products were denatured in NaOH and washed before a sequencing primer was annealed. The pyrosequencing reaction starts from the 3'-end of the sequencing primer. Nucleotides (A, T, C, and G) were dispensed into each sample well, one at a time. Whenever a base complementary to the base in the PCR product is added, it is incorporated into the growing DNA strand, resulting in an enzymatic cascade and production of light. The light intensity is measured at each dispensation and presented graphically in a pyrogram. The dispensation order was generated automatically by the Pyromark CpG Software 1.0.11 and modified according to recommendations by the provider (two dispensations added). The dispensation order was GTCG**C**TTAGTCTGTTCG**T**ATCAGTCGTCA (extra dispensations in bold). The extra C dispensation in the beginning of the sequence served as a bisulfite control. The additional T dispensation was included to remove background noise in the following CpG site, thus the peak was excluded as a reference peak in data analysis. The minimal signal value was set to 100 as recommended in the Pyromark CpG Software user manual. Apart from these changes, the results were analyzed using default software settings.

The pyrosequencing threshold was determined from the mean methylation value in the five analyzed CpG sites and the mean standard deviation (X + 2SD) in the four meningiomas. Glioma samples were scored as methylation positive by pyrosequencing if all five CpG sites had methylation values higher than the resulting threshold of 2.68%.

### MGMT expression analysis by real-time reverse transcriptase PCR

Total RNA was extracted from 81 of the 134 glioma samples. The tissue samples were stored frozen in RNAlater and total RNA was extracted using a standard TRIzol protocol. RNA quantity and integrity were examined using a NanoDrop ND-1000 Spectrophotometer (Thermo Fisher Scientific) and an Agilent BioAnalyzer 2100 (Agilent Technologies), respectively. Total RNA was reverse transcribed using cDNA by random hexamer primers and the High Capacity cDNA Reverse Transcription Kit (Applied Biosystems, Life Technologies) according to the manufacturers' protocol. The real-time PCR was carried out in triplicates in 384 well plates using the 7900HT Fast-Real time PCR machine (Applied Biosystems, Life Technologies). The total reaction volume was 20 μl and contained 20 ng cDNA, 1× TaqMan Universal PCR Mastermix (Applied Biosystems, Life Technologies), and 1× TaqMan Gene Expression assay (Applied Biosystems, Life Technologies; see below). The PCR was run at 50°C for 2 min, 95°C for 10 min, and 40 cycles of 95°C for 15 s and 60°C for 60 s. Each sample was analyzed with two different TaqMan Gene Expression Assays for *MGMT *(Hs01037698_m1 and Hs00172470_m1; part number 4331182, Applied Biosystems, Life Technologies) as well as two endogenous controls (*ACTB*; part number 4352935E, and *GUSB *part number 4333767 F, Applied Biosystems, Life Technologies). The median result in each triplicate was used for data analysis.

Each plate included several non-template controls and a standard curve generated by a serial dilution of total cDNA reverse transcribed from Universal Human Reference RNA (Stratagene, Agilent Technologies). Ct-values were determined automatically with the default settings in the software SDS 2.2.2 (Applied Biosystems, Life Technologies) and converted to quantity using the standard curves. Samples with a Ct-value above 35 were censored. The quantity was normalized by dividing the quantity of *MGMT *expression by the average quantity of *ACTB *and *GUSB*.

### Statistical analyses

Statistical analyses were performed in SPSS 16.0. (SPSS Inc.). We used McNemar's chi-squared test for comparison of the two methylation detection methods and Kruskal-Wallis' rank sum test to determine potential differences in methylation level between high- and low-grade gliomas. Overall survival analysis was performed using the Kaplan-Meier procedure and included all primary glioblastoma patients who were treated with standard radiotherapy (2 Gy × 30) and concomitant, and in some cases adjuvant, TMZ (n = 58). Patients' date of death was collected from the National Population Register. Survival was calculated from date of first surgery. Log-rank test was based on 24 months overall survival. T-tests for independent samples were performed to compare average gene expression of *MGMT *in samples with versus samples without a methylated *MGMT *promoter. All *P*-values < 0.05 were considered statistically significant.

## Results

### MGMT promoter methylation status

The results of the two methylation detection methods, qMSP and pyrosequencing, were compared (McNemar's chi-squared; *P *< .001). Using pyrosequencing, 66% of the samples were scored as methylation positive, whereas qMSP analysis resulted in 34% positive samples. The percentage of methylated samples, the interquartile range of the PMR values, and pyrosequencing results are summarized in Table 2. All glioma samples scored as methylated by qMSP were also found methylated by pyrosequencing (n = 46). Likewise, another 46 glioma samples scored as unmethylated by qMSP were also found unmethylated by pyrosequencing. A total of 42 glioma samples were scored as methylated by pyrosequencing and unmethylated by qMSP. All samples with PMR values above zero in one of the two qMSP assays (n = 16) were detected as methylation positive by pyrosequencing. The methylation levels (amount of methylation) determined by pyrosequencing and the MGMT_1 qMSP assay were significantly lower in methylated low-grade gliomas compared to methylated high-grade gliomas, *P *= .003 and *P *= .018, respectively. However, the difference was not statistically significant when testing methylation levels determined by the MGMT qMSP assay (*P *= .208).

**Table 2:** *MGMT *methylation frequencies and methylation level in methylated glioma samples

Group	qMSP results			Pyrosequencing results	
	
		Median and IQR			Median and IQR
				
		calculated from PMR			calculated from
				
	Methylated samples	MGMT assay	MGMT_1 assay	Methylated samples	mean CpG methylation
**Low-grade gliomas**	**9/35** **(25.7%)**	**3.30** **(0.78-19.66)**	**0.50** **(0.29-2.57)**	**34/35** **(97.1%)**	**16.98** **(10.97-38.29)**

Astrocytoma WHO grade II	2/10 (20.0%)	1.19 (0.85-1.54)	0.30 (0.29-0.30)	10/10 (100.0%)	12.56 (7.24-16.68)

Oligodendroglioma WHO grade II	1/6 (16.7%)	0.78 -	0.87 -	5/6 (83.3%)	29.51 (24.23-33.17)

Oligoastrocytoma WHO grade II	6/17 (35.3%)	11.72 (3.42-24.16)	1.54 (0.32-7.83)	17/17 (100.0%)	24.90 (14.41-47.52)

Low-grade neuroepithelial tumours (not otherwise specified)	0/2 (0.0%)	-	-	2/2 (100.0%)	6.83 (5.33-8.32)

**High-grade gliomas**	**37/99** **(37.4%)**	**9.18** **(4.39-17.81)**	**5.59** **(1.89-12.37)**	**54/99** **(54.5%)**	**47.14** **(18.50-62.87)**

Anaplastic astrocytoma WHO grade III	1/4 (25.0%)	24.19 -	22.35 -	2/4 (50.0%)	55.82 (52.95-58.70)

Anaplastic oligodendroglioma WHO grade III	6/6 (100.0%)	19.30 (7.71-54.53)	11.61 (6.68-16.09)	6/6 (100.0%)	70.85 (52.98-82.52)

Anaplastic oligoastrocytoma WHO grade III	1/3 (33.3%)	17.81 -	0.90 -	2/3 (66.7%)	52.99 (48.03-57.94)

Glioblastoma WHO grade IV	29/86 (33.7%)	7.98 (4.00-14.55)	5.39 (1.89-11.37)	44/86 (51.2%)	43.31 (12.05-61.04)

*Primary glioblastoma*	27/80 (33.8%)	8.67 (3.85-15.33)	5.59 (1.80-11.48)	40/80 (50.0%)	40.68 (11.12-59.05)

*Included in survival analysis*^a^	19/58 (32.8%)	5.53 (2.58-10.42)	3.22 (1.14-10.26)	29/58 (50.0%)	34.88 (10.64-48.73)

*Secondary glioblastoma*	2/6 (33.3%)	5.76 (5.18-6.34)	2.75 (2.41-3.08)	4/6 (66.7%)	55.26 (42.67-63.93)

**All glioma samples**	**46/134** **(34.3%)**	**8.33** **(3.40-18.55)**	**4.36** **(0.88-11.54)**	**88/134 (65.7%)**	**34.03** **(12.42-54.83)**

*Abbreviations: PMR*, Percentage of methylated reference, *qMSP*, quantitative methylation specific PCR, *IQR*, interquartile range

a)Primary glioblastoma patients treated with standard radiation and at least concomitant temozolomide

### MGMT promoter methylation status and survival of primary glioblastoma patients

**Figure 1:**
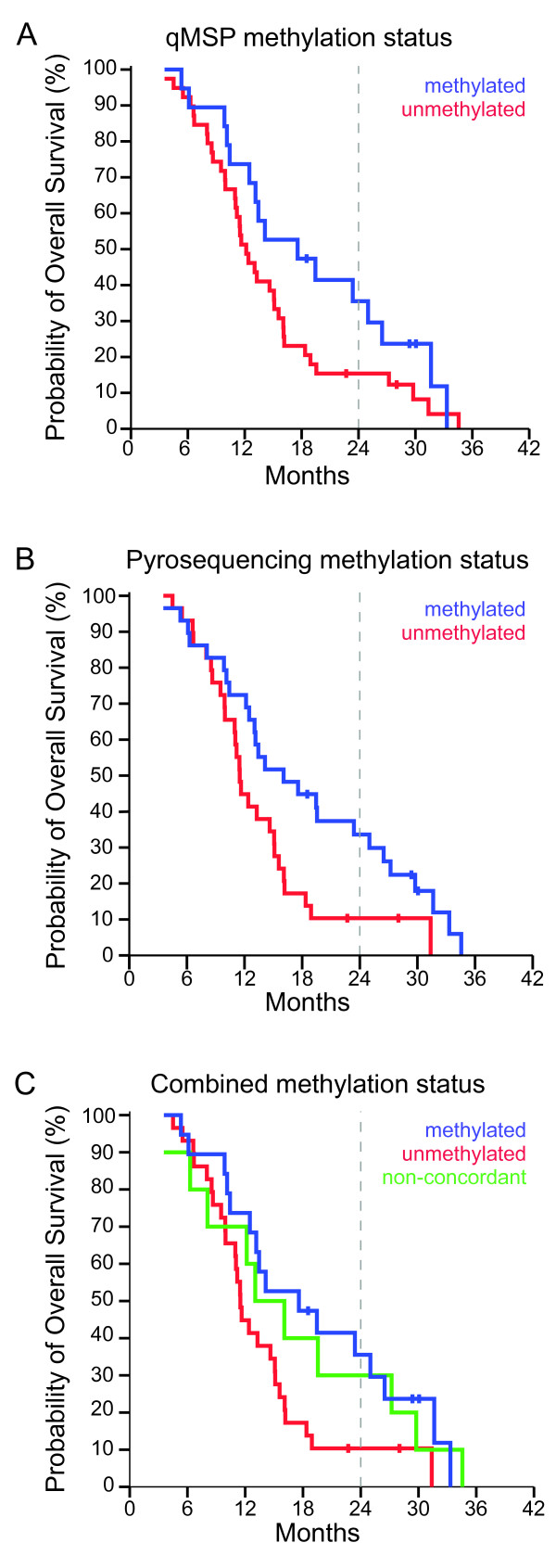
**Overall survival in primary glioblastoma patients treated with standard radiotherapy and concomitant temozolomide**. (**A**) Methylation status based on results from qMSP. Blue line; methylated, red line; unmethylated. (**B**) Methylation status based on results from pyrosequencing. Blue line; methylated, red line; unmethylated. (**C**) Methylation status based on both qMSP and pyrosequencing results. Blue line; methylated in both methods, red line; unmethylated in both methods, green line; methylated in pyrosequencing assay and unmethylated in qMSP assay. Abbreviation: qMSP, quantitative methylation specific PCR

Regardless of the method used to determine methylation status, the 24-months overall survival curves for primary glioblastoma patients receiving standard radiotherapy and at least concomitant TMZ displayed a trend towards better survival in the patient group with methylated *MGMT *promoter than in the patient group with unmethylated *MGMT *promoter (log-rank test, *P *= .06 and *P *= .02 for qMSP and pyrosequencing, respectively; Figure 1A and 1B). Median overall survival in the patient group with methylated *MGMT *promoter was about 5 months longer than the median overall survival for patients with an unmethylated *MGMT *promoter. Patient characteristics and results from the overall survival analysis are summarized in Table 3. Overall survival for patients with conflicting *MGMT *promoter methylation results as assessed by qMSP and pyrosequencing was intermediate when compared to the two groups of patients with concordant results (Figure 1C).

**Table 3:** Patient characteristics and results of survival analysis for primary glioblastoma patients when stratified by *MGMT *methylation status

Overall survival		MGMT methylation status			
	
	Total	qMSP		Pyrosequencing	
		
		Methylated	Unmethylated	Methylated	Unmethylated
Patients (n)	58	19	39	29	29

Male/Female	31/27	10/9	21/18	15/14	16/13

Mean age^a^ (Standard deviation)	58.5 (9.1)	57.5 (8.7)	59.0 (9.3)	59.3 (8.5)	57.7 (9.7)

Median survival^b^ (Standard error)	13.1 (1.1)	17.6 (4.2)	12.2 (1.0)	16.1 (3.7)	11.5 (0.4)

2-years overall survival (%) (Standard error)	21.8 (0.06)	35.5 (0.11)	15.4 (0.06)	33.6 (0.09)	10.3 (0.06)

a)Years; b) Months

### MGMT gene expression in samples with and without MGMT promoter methylation

**Figure 2:**
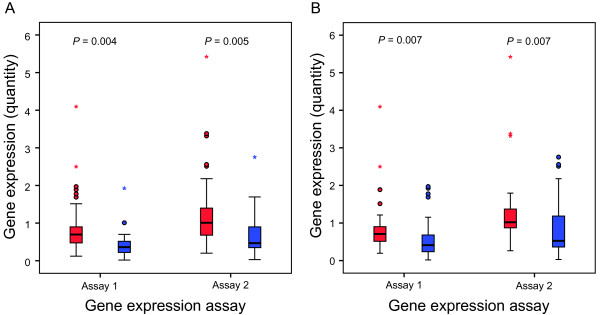
**Gene expression level of *MGMT *is associated with promoter DNA methylation status**. *MGMT *gene expression in methylated (blue box plots) and unmethylated (red box plots) tissue samples analyzed by two different primer/probe sets (Assay 1; Hs00172470_m1 and assay 2; Hs01037698_m1). (**A**) Methylation status based on results from qMSP. (**B**) Methylation status based on results from pyrosequencing. Abbreviation: qMSP, quantitative methylation specific PCR

**Figure 3:**
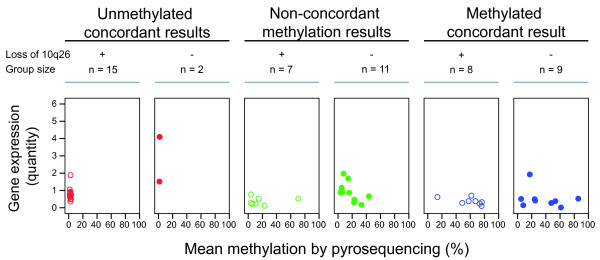
**Scatter plots of *MGMT *gene expression quantity in methylated and unmethylated samples**. Red color indicates samples with negative methylation status by qMSP and pyrosequencing, blue color indicates samples with positive methylation status by qMSP and pyrosequencing, and green color indicate samples with non-concordant methylation status in the qMSP and pyrosequencing analysis. Circles indicate samples with loss of 10q26, whereas dots represent samples without loss of this region. Plots are based on the normalized gene expression detected by primer/probe set Hs00172470_m1. The plots based on the normalized gene expression detected by the other primer/probe set, Hs01037698_m1, were similar (data not shown). Abbreviation: qMSP, quantitative methylation specific PCR

The average gene expression value in samples with methylated *MGMT *promoter was significantly lower than the average gene expression value in samples with unmethylated *MGMT *promoter (*P *< 0.01) regardless of methylation detection method (Figure 2). The difference was statistically significant also after elimination of the most evident outlier. The scatter plots (Figure 3) show the gene expression level in samples where we also had access to karyotypic and/or CGH data [16,17] (author H-S. S. Dahlback, unpublished data) (n = 52).

## Discussion

Reported frequencies of *MGMT *promoter methylation in subgroups of gliomas vary considerably, as shown in Additional file 1: Tables S1 and S2. Various methylation detection methods and different primer sets and threshold values have been used. In the present study, we report *MGMT *promoter methylation frequencies in gliomas determined by qMSP (low-grade gliomas 26% and high-grade gliomas 37%) as well as by pyrosequencing (low-grade gliomas 97% and high-grade gliomas 55%). It should be noted that the CpG sites interrogated in the qMSP and pyrosequencing assays are only partially overlapping (Additional file 1: Figure S1), and caution should therefore be made when directly comparing the results. The *MGMT *promoter is typically reported methylated in 30-60% of glioblastomas [11] and in 30-90% of low-grade gliomas [12,18]. Compared to these reports, our qMSP methylation frequencies are in the lower range whereas the pyrosequencing methylation frequencies are in the upper range.

The vast majority of previous studies of *MGMT *promoter methylation in gliomas have used gel based methylation-specific PCR (MSP), which is a qualitative and time-consuming method. The manual methylation scoring based on interpretation of gel band intensities will vary in stringency level between labs, which, in addition to the use of different primers, may in part explain some of the observed difference in results observed from MSP based studies. This is exemplified by two works using the same primer sets but reporting very different methylation frequencies of 23% and 44% in newly diagnosed glioblastoma samples [19,20]. In contrast to MSP, qMSP is a quantitative, standardized, high-throughput method which is easy to perform and the results are easy to evaluate. Thus, the method is more suitable for use in routine testing. To detect methylation by amplification of methylated alleles in MSP and qMSP, all CpG sites on the same DNA strand covered by the primers have to be methylated. Compared with traditional MSP, qMSP is even more conservative as it includes a methylation-specific probe and thereby typically covers more CpG sites that all have to be methylated. This may lower the sensitivity of the assay, but more importantly, increases the specificity, as underscored by Parella et al. who analyzed *MGMT *promoter methylation using both MSP and qMSP assays. In glioma samples the two methods showed good concordance, whereas the results from normal brain samples demonstrated that MSP may hold a higher risk of false positive results [21]. In the present study, we used two overlapping qMSP assays and only scored samples as methylated if they had a positive PMR value in both assays. The results from the two assays were generally overlapping. Of the 134 analyzed gliomas samples only 16 of them had conflicting methylation status from the qMSP assays. The PMR-values seemed to be somewhat higher in one of the qMSP assays. This is probably a result of the inclusion of different CpG sites in the two assays (Additional file 1: Figure S1). The conservative nature of the qMSP assay may explain why our qMSP methylation frequencies are in the lower range of previously reported MSP results [11,12,18]. One of the primer/probe sets used in the present study corresponds to the assay used by Parrella and coworkers [13,21]. The second qMSP assay is modified after a recent publication by Rivera et al. [14] who found *MGMT *promoter methylation in 24% of GBM patients, which is similar to the methylation frequency found by us.

As expected, the *MGMT *promoter methylation frequencies as measured by pyrosequencing were found to be in the upper range compared to previous MSP based findings. In contrast to MSP and qMSP, the pyrosequencing technique is able to detect low levels of methylation because methylation in each CpG site is measured independently of the methylation status in surrounding CpG sites. Indeed, our methylation frequency in high-grade gliomas (55%) is highly concordant with the GBM methylation frequency recently determined in a pyrosequencing work by Dunn et al. (53%) [22].

Choice of threshold values for scoring samples as methylation positive or not may also explain some of the differences observed in reported methylation frequencies. Ideally, the threshold value should be determined using a test series of a large number of normal tissue samples as well as tumor samples. The threshold value can thereafter be chosen to give a high sensitivity (with the risk of producing false positives) or a high specificity (with the risk of failing to identify all positive cases as such). We have used high-quality DNA extracted from fresh frozen tissue for all methylation analyses. In some neurooncology centers sampling of fresh frozen tissue is not a standard procedure, hence formalin-fixed paraffin embedded (FFPE) tissue is a frequently used DNA source. All *MGMT *promoter fragments amplified in the present study are short (qMSPs 83-119 bp and pyrosequencing ~100 bp) and will most likely be amplifiable also in DNA extracted from FFPE tissue. Lacking access to normal brain tissue, we used four benign meningioma samples to set the threshold values for scoring samples as methylation positive. The threshold values were determined so that all the meningiomas were scored as methylation negative. These benign tumors showed little (pyrosequencing, mean methylation range 1.39-1.55%) to no (qMSP, PMR 0% in both assays) *MGMT *promoter methylation, resulting in low threshold values, thus supporting the assumption that meningiomas are suitable alternatives to normal tissue samples for threshold determination. However, this should be confirmed by validation studies in independent sample series. Brain tissue from surgery in epileptic patients is an alternative to the meningioma tissue for establishing cutoff values.

In accordance with previous reports, our results show that the overall survival for patients with a methylated *MGMT *promoter is better than for patients with an unmethylated promoter [10,20,23,24]. The observed difference at 24 months was significant based on the pyrosequencing results but only borderline significant based on the qMSP results. The log-rank test results indicate that both methylation detection methods are able to identify primary glioblastoma patients with a somewhat better prognosis. However, the survival curve differences are more distinct when using the pyrosequencing based methylation status, implying that this is the better method to use for estimating the prognosis. These results are in line with the observation that the patients with non-concordant methylation findings (unmethylated by qMSP and methylated by pyrosequencing) showed a trend towards better survival than patients with unmethylated *MGMT *promoter by both methods (Figure 1C). The last mentioned finding should, however, be validated in an independent sample set. Nevertheless, based on the observations done here, it could be argued that a primary glioblastoma should be regarded *MGMT *promoter methylated if the pyrosequencing result is positive. Because all glioblastoma patients receive TMZ as part of the Stupp regimen, the methylation status of the *MGMT *promoter does not change the therapeutic regime today. Nonetheless, it is a prognostic marker [11] of clinical interest and may be relevant for evaluation of pseudoprogression [24]. It is also interesting that, independent of the method used, the methylation level (amount of methylation) observed in methylation positive low-grade gliomas is low compared to the level observed in methylation positive high-grade gliomas. This has not been reported previously and may in part explain the large difference in methylation frequency as assessed by qMSP and pyrosequencing in low-grade gliomas. The clinical relevance of this finding remains to be determined and the data should be validated in an independent data set. Nevertheless, the overall survival analysis, which includes GBM with a low methylation level, suggests that pyrosequencing is the better method for predicting prognosis in primary GBM patients. This may also suggest an advantage of a low methylation level in low-grade gliomas. There are not many studies reporting *MGMT *promoter methylation frequencies in large series of low-grade gliomas. However, two studies analyzing 68 and 185 low-grade gliomas report methylation frequencies of 93% and 81%, respectively, using the same MSP primers in a nested two-stage approach [18,25]. These frequencies match our frequency (97%) detected by the sensitive pyrosequencing approach. However, other studies with smaller sample series report lower frequencies in the range 40-50% [26-29] when analyzed by conventional MSP.

We found a significant association between *MGMT *promoter methylation and reduced gene expression, regardless of methylation detection method and gene expression assay used. Based on this, one could suggest that the gene expression level might be analyzed instead of promoter methylation. However, the gene expression level in methylated and unmethylated samples shows considerable overlap (Figure 2) which may be due to lack of a linear relationship between the region analyzed for promoter methylation and gene expression. The most commonly analyzed region in the *MGMT *gene promoter covers 9 of the totally 97 CpG sites in the promoter, and it has been suggested that methylation in some specific CpG sites correlates better with reduction in gene expression level than analysis of the common MSP region (Additional file 1: Figure S1) [30]. On the other hand, factors such as contamination with normal cells and loss of one *MGMT *allele may also influence the detected gene expression level. For a subset of the samples with gene expression data and methylation status, we had access to karyotypic and/or CGH data [16,17] (author H-S. S. Dahlback, unpublished data). Figure 3 illustrates that the *MGMT *gene expression seemed to be affected by loss of the 10q26 chromosome band. Interestingly, but not unexpectedly, the loss of this chromosome band seemed to have a larger impact on gene expression in samples with a low methylation level (mean methylation by pyrosequencing < 20%) compared to highly methylated samples. However, these observations are based on results from small groups and should be tested in a larger dataset.

It is important to keep in mind that it is the MGMT protein that counteracts the effect of TMZ by removing methyl adducts at the O^6^-position of guanine. A recent study using human tumor cell lines derived from glioblastomas and other tumors concluded that the response to TMZ is better predicted by MGMT protein expression than by promoter methylation status [31]. However, although cancer cell lines are useful models for the in vivo situation, findings should be validated in patient sample series, and so far immunohistochemical analyses of the MGMT protein level in human tumor samples have been inconclusive when correlated with patient outcome [32].

## Conclusions

Taken together, our findings corroborate earlier conclusions that *MGMT *promoter methylation is of prognostic value for primary glioblastoma patients [9,10,20,22,33], and this status is of interest for the patients, their relatives, and treating physicians. Therefore, in our opinion determination of *MGMT *promoter methylation status should be incorporated into standard management programs for patients with GBM. There is currently no "gold standard" for which technique to use for assessing clinically meaningful *MGMT *promoter methylation. In this study pyrosequencing came across as a slightly better method than qMSP when looking at the prognostic value of *MGMT *promoter methylation status in primary glioblastomas. Both qMSP and pyrosequencing are easy to perform and high-throughput methods; the choice of method therefore becomes one based on utilitarian considerations.

## Competing interests

The authors declare that they have no competing interests.

## Authors' contributions

Authors GEL, PB, RAL, and SH were responsible for the study design. EH and TRM were responsible for collecting clinical samples. H-SSD collected and registered the patient consents. ABH, H-SSD, and PB collected and organized the clinical data. DS reviewed the histological diagnoses. Authors ABH, HH, GEL, and MH contributed in the laboratory work. ABH, GEL, HH, PB, RAL, and SH contributed in the interpretation of the data. ABH drafted the manuscript and made the tabular literature summary included in the Additional file 1. All authors carefully revised the manuscript and approved the final version.

## Supplementary Material

Additional file 1 **Supplementary material **[8-10,12-14,18-30,34-120]: CpG sites in the *MGMT *promoter frequently analyzed for DNA methylation. Description of the criteria for inclusion. Studies of *MGMT *promoter methylation in high-grade gliomas-summary of methylation frequencies and methodological details. Studies of *MGMT *promoter methylation in low-grade gliomas-summary of methylation frequencies and methodological details. Click here for file
