# Supplementary material for: MGMT promoter methylation in gliomas-assessment by pyrosequencing and quantitative methylation-specific PCR
Source: J Transl Med. 2012 Mar 6;10:36. doi: 10.1186/1479-5876-10-36 (PMC3311573; doi:10.1186/1479-5876-10-36)
Supplement: Additional file 1 — Supplementary material [8-10,12-14,18-30,34-120]: CpG sites in the MGMT promoter frequently analyzed for DNA methylation. Description of the criteria for inclusion. Studies of MGMT promoter methylation in high-grade gliomas-summary of methylation frequencies and methodological details. Studies of MGMT promoter methylation in low-grade gliomas-summary of methylation frequencies and methodological details. [file 1479-5876-10-36-S1.DOC]

**Supplementary material**

**Content:**

- **Supplementary figure 1:**

CpG sites in the *MGMT* promoter frequently analyzed for DNA methylation

- **Introduction to Supplementary tables 1 and 2**Description of the criteria for inclusion
- **Supplementary table 1:**

Studies of *MGMT* promoter methylation in high-grade gliomas
- summary of methylation frequencies and methodological details

- **Supplementary table 2:**

Studies of *MGMT* promoter methylation in low-grade gliomas
- summary of methylation frequencies and methodological details

**Supplementary figure 1:**


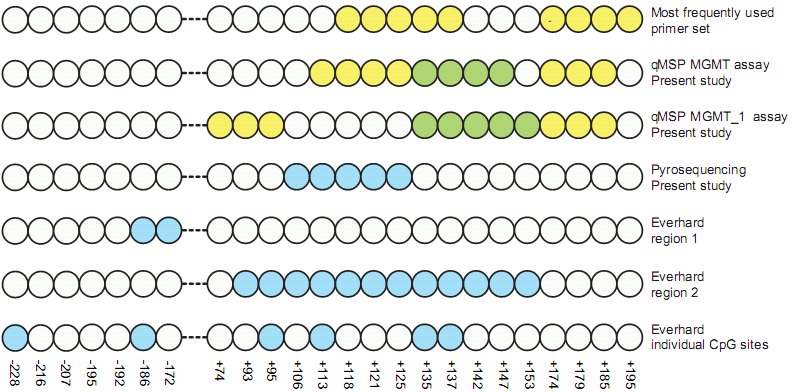


**SUPPLEMENTARY FIGURE 1**: CpG sites in the *MGMT* promoter frequently analyzed for DNA methylation. The illustration indicates CpG sites analyzed in the present study and in the traditional MSP assay as reported by Esteller et al. in 1999 [1], as well as individual CpG sites and regions with a high correlation to gene expression level as reported by Everhard et al. in 2009 [2]. Yellow color indicates CpG sites covered by primers whereas green color indicates CpG sites covered by probes. Blue color indicates CpG sites analyzed by pyrosequencing. Abbreviations: MSP, methylation specific PCR; qMSP, quantitative methylation specific PCR.

**Introduction to Supplementary tables 1 and 2:**

Relevant publications to include in a tabular summary of the reported *MGMT* promoter methylation frequencies in gliomas were found by a standard search in the PubMed database, entering the words “gliomas”, “MGMT”, and “methylation” in the search box. The search was restricted to only include publications in English published before January 1^st^ 2011. Search details were as follows: (("glioma"[MeSH Terms] OR "glioma"[All Fields]) AND mgmt[All Fields] AND ("methylation"[MeSH Terms] OR "methylation"[All Fields])) AND (English[lang] AND ("1"[PDAT] : "2010/12/31"[PDAT])).

Publications included in the summary reported *MGMT* promoter methylation status in at least 20 cases of high- and/or low-grade gliomas with astrocytic and/or oligodendroglial differentiation in adult patients. We also included two other relevant publications that were not part of the search result in PubMed, but otherwise fulfilled the inclusion criteria [1, 3] Publications that met the described inclusion criteria are listed in Supplementary tables 1 and 2

It should be noted that the studies included in this extensive tabular summary have different sample inclusion criteria and to some extent use different detection methods and thresholds. Hence, caution should be exerted when comparing the promoter methylation frequencies between individual studies.

| **SUPPLEMENTARY TABLE 1:** Studies of *MGMT* promoter methylation in high-grade gliomas - summary of methylation frequencies and methodological details. | | | | | | |
| --- | --- | --- | --- | --- | --- | --- |
| **High-grade glioma studies** | | | | | | |
| **First author (year) (reference)** | **Total methylation frequency %** | **Subgroup**  **methylation frequency^a^** | **Diagnosis** | **Tissue** | **Method** | **Methodological details** |
| Ang  (2010) [4] | 54% (57/105) | - | Glioblastoma (GBM), newly diagnosed,  WHO grade IV | FFPE | MSP | Conventional MSP primers.^b^ |
| Balaña  (2003) [5] | 38% (8/21) | - | Glioblastoma (GBM),  WHO grade IV | Frozen | MSP | Conventional MSP primers.^b^ |
| Blanc  (2004) [6] | 68% (30/44) | - | Glioblastoma (GBM),  WHO grade IV | Frozen | MSP | Conventional MSP primers.^b,c^ |
| Brandes  (2006) [7] | 46% (10/22) | - | Glioblastoma (GBM), recurrent,  WHO grade IV | FFPE | MSP  Nested two-stage approach | Conventional primers for nested two-stage approach^d^, some cases amplified with optional first step primers.^e^ |
| Brandes  (2006) [8] | 69% (37/54) | 30% (X/X) | Anaplastic oligoastrocytoma (AOA),  WHO grade III | FFPE | MSP  Nested two-stage approach | Conventional primers for nested two-stage approach^d^, some cases amplified with optional first step primers.^e^ |
|  |  | 70% (X/X) | Anaplastic oligodendroglioma (AO),  WHO grade III |  |  |  |
| Brandes  (2008) [9] | 35% (36/103) | - | Glioblastoma (GBM), newly diagnosed,  WHO grade IV | FFPE | MSP  Nested two-stage approach | Conventional primers for nested two-stage approach^d^, some cases amplified with optional first step primers.^e^ |
| Brandes  (2009) [10] | 34% (32/95) | - | Glioblastoma (GBM), newly diagnosed,  WHO grade IV | FFPE | MSP  Nested two-stage approach | Conventional primers for nested two-stage approach^d^, some cases amplified with optional first step primers.^e^ |
| Brandes  (2009) [11] | 43% (16/37) | - | Glioblastoma (GBM), newly diagnosed, patients ≥ 65 years,  WHO grade IV | FFPE | MSP  Nested two-stage approach | Conventional primers for nested two-stage approach^d^, some cases amplified with optional first step primers.^f^ |
| Brandes  (2010) [12] |  | 34% (13/38) | Glioblastoma (GBM), newly diagnosed,  first surgery,  WHO grade IV | FFPE | MSP  Nested two-stage approach | Conventional primers for nested two-stage approach^d^, some cases amplified with optional first step primers.^e^ |
|  |  | 29% (11/38) | Glioblastoma (GBM), recurrence,  second surgery,  WHO grade IV |  |  |  |
| Brell  (2005) [13] | 50% (20/40) | 56% (18/32) | Anaplastic astrocytoma (AA),  WHO grade III | FFPE  and  Frozen | MSP | Conventional MSP primers.^b,c^ |
|  |  | 25% (2/8) | Anaplastic oligodendroglioma (AO) and anaplastic oligoastrocytoma (AOA),  WHO grade III |  |  |  |
| Cancer Genome Atlas Research Network (2008) [14] | 21% (19/91) | - | Glioblastoma (GBM), newly diagnosed,  WHO grade IV | Frozen | Bead array | The DNA methylation values (β –values) was calculated, scaled (β’), and criteria for scoring samples as partially methylated and fully metylated was established by default. Partially methylated samples β’ > 0.25, fully methylated (β’ > 0.75). |
| Cankovic  (2007) [15] | 49% (60/123) | 44% (35/80) | Glioblastoma (GBM),  WHO grade IV | FFPE | MSP  two-step approach | Conventional MSP primers.^b,c^ |
|  |  | 38% (3/8) | Anaplastic astrocytoma (AA),  WHO grade III |  |  |  |
|  |  | 63% (19/30) | Anaplastic oligodendroglioma (AO),  WHO grade III |  |  |  |
|  |  | 50% (2/4) | Mixed anaplastic oligodendroglioma + astrocytoma (AOA),  WHO grade III |  |  |  |
|  |  | 100% (1/1) | Oligodendroglioma + anaplastic astrocytoma (O+AA),  WHO grade III |  |  |  |
| Cao  (2000) [16] | 61 % (46/76) | - | Glioblastoma (GBM),  WHO grade IV | FFPE  and Frozen | MSP | Conventional MSP primers.^b^ |
| Christmann  (2010) [17] | 37% (25/68), MSP-P1  31% (21/68), MSP-P2 | 39% (18/46), MSP-P1  33% (15/46), MSP-P2 | Glioblastoma (GBM), newly diagnosed,  WHO grade IV | FFPE | MSP | MSP-P1 using conventional MSP primers^b^.  MSP-P2 using primers for metylated MGMT, and β-actin reaction for normalization. Primers reported. (MGMT methylated forward primer is identical with the corresponding forward primer in the conventional MSP assay). The methylation status was determined in relation to the signal obtained for the β-actin promoter. Samples was considered to be methylated if the result indicated >20% methylation. |
|  |  | 32% (7/22), MSP-P1  27% (6/22), MSP-P2 | Anaplastic astrocytoma (AA) and anaplastic oligoastrocytoma (AOA), newly diagnosed,  WHO grade III |  |  |  |
|  | - | 5% (1/19^g^), MSP-P1  11% (2/19^g^), MSP-P2 | Glioblastoma (GBM), recurrent,  WHO grade IV |  |  |  |
| Clarke  (2009) [18] | 19% (9/48) | - | Glioblastoma (GBM), newly diagnosed,  WHO grade IV | - | MSP^h^ | OncoMethylome Sciences, Inc. |
| Costa  (2010) [19] | 56% (24/43) | - | Glioblastoma (GBM), newly diagnosed,  WHO grade IV | FFPE | MSP  Nested two-stage approach | Conventional primers for nested two-stage approach ^d^ |
| Costa  (2010) [20] | 48% (38/80) | - | Glioblastoma (GBM), newly diagnosed,  WHO grade IV | FFPE | MSP  Nested two-stage approach | Conventional primers for nested two-stage approach.^d^ |
| Crinière  (2007) [21] | 58% (126/219) | - | Glioblastoma (GBM), primary,  WHO grade IV | Frozen | MSP  Nested two-stage approach | Conventional primers for nested two-stage approach.^c,d^ |
| Drabycz  (2010) [22] | 53% (31/59^i^)  50% (36/72^i^) | - | Glioblastoma (GBM), newly diagnosed,  WHO grade IV | FFPE | MSP  Nested two-stage approach | Conventional primers for nested two-stage approach.^d^ |
| Ducray  (2010) [23] | 21% (6/28) | - | Glioblastoma (GBM), newly diagnosed  WHO grade IV | Frozen | MSP  Nested two-stage approach | Conventional primers for nested two-stage approach.^c,d^ |
| Dunn  (2009) [24] | 53% (58/109) | - | Glioblastoma (GBM), newly diagnosed  WHO grade IV | Smears, FFPE  and  Frozen | Pyrosequencing 12 CpG sites | Samples considered to be methylated it the average methylation level was ≥ 9% ) in more than one independent bisulfite modification. Cut-off determined using DNA from six non-neoplastic brain samples (mean ± 2SD). |
| El Hindy  (2010) [25] | 23% (24/103) | - | Glioblastoma (GBM), primary,  WHO grade IV | FFPE | MSP | Primers as reported by Wick *et al.* (2007) [26]. |
| Eoli  (2007) [27] | 48% (41/86) | 42% (30/72) | Glioblastoma (GBM), primary,  WHO grade IV | - | MSP | Conventional MSP primers^b^ labeled with fluorescent. PCR products were examined automatically and quantitated. The ratio of the peak height in the methylated and unmethylated reaction was calculated. Samples was considered to be methylated if the peak height ratio was >0.1. |
|  |  | 79% (11/14) | Glioblastoma (GBM), secondary,  WHO grade IV |  |  |  |
| Esteller  (1999) [1] | 43% (46/107) | 50% (10/ 20) | Anaplastic astrocytoma (AA),  WHO grade III | - | MSP | Conventional MSP primers.^b^ |
|  |  | 41% (36/ 87) | Glioblastoma (GBM),  WHO grade IV |  |  |  |
| Esteller  (2000) [28] | 40% (19/47) | 41% (12/29) | Glioblastoma (GBM), newly diagnosed,  WHO grade IV | Frozen | MSP | Conventional MSP primers.^b^ |
|  |  | 39% (7/18) | Anaplastic astrocytoma (AA), newly diagnosed,  WHO grade III |  |  |  |
| Etcheverry  (2010) [29] | 60% (30/50) | - | Glioblastoma (GBM), newly diagnosed  WHO grade IV | Frozen | Bead chip | The DNA methylation values (β –values) was calculated and CpG sites were identified as methylated/unmethylated acording to the β cut-off value 0.10. This threshold was chosen as it resulted in the best stratification (p-value) according to the log-rank test. |
| Everhard  (2009) [2] | 67% (36/54) | - | Glioblastoma (GBM),  WHO grade IV | Frozen | Pyrosequensing 52 CpG sites | Sample results were dichotomized. The used cut-off was 10.65% in all 52 CpG sites and was determined using DNA from 24 non-neoplastic brain samples (mean ± 2SD). |
| Fabi  (2010) [30] | 45%  (14/31) | - | Malignant gliomas, recurrent:  Anaplastic astrocytoma (A), anaplastic oligodendroglioma (AO), WHO grade III and Glioblastoma (GBM),  WHO grade III | FFPE | MSP  two-step approach | Primers not reported. |
| Felsberg  (2009) [31] | 39% (22/66) | - | Glioblastoma (GBM), primary,  WHO grade IV | FFPE  and Frozen | MSP | Primers as reported by Wick *et al.* (2007) [26]. |
| Gan  (2010) [32] | 48% (10/21)  both methods | 100% (7/7)  both methods | Anaplastic oligodendroglioma (AO),  WHO grade III | FFPE | MALDI-TOF MS  8 CpG units  20 CpG sites  and  MS-HMR | MALDI-TOF MS, samples was scored as methylated if the peak height ratio was higher than the calculated cut-off value in ≥4 CpG units. Cut-off determined using DNA from 4 normal control periperheral blood samples (mean ± 2SD).  MS-HRM, a series of standards from fully methylated human DNA was used to quantify the relative methylation in the sample. Samples were scored as methylated if the methylation score was ≥ 10%. |
|  |  | 21% (3/14)  both methods | Anaplastic oligoastrocytoma (AOA),  WHO grade III |  |  |  |
| Gerstner  (2009) [33] | 58% (37/64) | - | Glioblastoma (GBM), newly diagnosed, patients ≥ 70 years,  WHO grade IV | FFPE | MSP | Conventional MSP primers.^b^ |
| Glas  (2009) [34] | 48% (11/23) | - | Glioblastoma (GBM), newly diagnosed,  WHO grade IV | - | MSP | Conventional MSP primers^b^ or primers as reported by Wick *et al.* (2007) [26]. Not specified. |
| Gonzalez-Gomez  (2003) [35] | 63% (40/64) | 71% (15/21) | Anaplastic astrocytoma (AA),  WHO grade III | Frozen | MSP | Conventional MSP primers.^b^ |
|  |  | 54% (18/33) | Glioblastoma (GBM), primary,  WHO grade IV |  |  |  |
|  |  | 70% (7/10) | Glioblastoma (GBM), secondary,  WHO grade IV |  |  |  |

| Grasbon-Frodl  (2007) [36] | 40% (10/25)  total  30% (6/20),  in primary group  80% (4/5),  in secondary group | 25% (1/4) | Anaplastic astrocytoma (AA), primary,  WHO grade III | Frozen | MSP  and  BS  25 CpG sites | MSP using conventional MSP primers.^b^  BS using primers as described by Möllemann 2005 [37]. A CpG site was considered methylated If the cytosine and thymine peaks were equally sized or the cytosine peak being larger. Samples were scored as methylated when more than 13 CpG sites were found to be methylated. 100% concordant results in MSP and BS. |
| --- | --- | --- | --- | --- | --- | --- |
|  |  | 31% (5/16) | Glioblastoma (GBM), primary,  WHO grade IV |  |  |  |
|  |  | 67% (2/3) | Anaplastic astrocytoma (AA), secondary,  WHO Grade III |  |  |  |
|  |  | 100% (2/2) | Glioblastoma (GBM), secondary,  WHO Grade IV |  |  |  |
| Grossman  (2009) [38] | 31% (9/29) | 29% (7/24) | Glioblastoma (GBM), newly diagnosed, patients ≤ 70 years,  WHO grade IV | FFPE | MSP^g^ | OncoMethylome Sciences, Inc. |
|  |  | 40% (2/5) | Glioblastoma (GBM), newly diagnosed, patients >70 years,  WHO grade IV |  |  |  |
| Hegi  (2004) [39] | 68% (26/38) | - | Glioblastoma (GBM), newly diagnosed,  WHO grade IV | FFPE  and  Frozen | MSP | Conventional MSP primers.^b^ |
| Hegi  (2005) [40] | 45% (92/206) | - | Glioblastoma (GBM), newly diagnosed,  WHO grade IV | FFPE | MSP  Nested two-stage approach | Conventional primers for nested two-stage approach.^d^ |
| Hervouet  (2009) [41] | 43% (23/53) | 33% (9/27) | Glioblastoma (GBM),  WHO grade IV  5 mC below median value | Primary cultured tumor cells | MSP | Conventional MSP primers.^b^  Experiments performed before passage 3 to 8. 5 mC value assessed by ELISA assay. |
|  |  | 52% (14/26) | Glioblastoma (GBM),  WHO grade IV  5 mC above median value |  |  |  |
| Ishii  (2007) [42] | 57% (25/44) | 40% (8/20) | Glioblastoma (GBM), recurrent,  WHO grade IV | Frozen | MSP | Conventional MSP primers.^b, c^ |
|  |  | 69% (11/16) | Anaplastic astrocytoma (AA), recurrent,  WHO grade III |  |  |  |
|  |  | 75% (6/8) | Anaplastic oligoastrocytoma (AOA), recurrent,  WHO grade III |  |  |  |
| Jesien-Lewandowicz  (2009) [43] | 72% (23/32) | - | Glioblastoma (GBM), primary,  WHO grade IV | Frozen | MSP  Nested two-stage approach | Conventional primers for nested two-stage approach.^d^ |
| Jeuken  (2007) [44] | 67% (28/42), MSP  58% (28/48), MS-MLPA | 100% (4/4), MSP  100% (5/5), MS-MLPA | Anaplastic oligodendroglioma (AO),  WHO grade III | FFPE  and  Frozen | MSP  MS-MLPA | MSP using conventional MSP primers^b^  (not dichotomized in publication^j^).  MS-MLPA using 3 MLPA probes. The methylation ratio for each sample was calculated (‘probe fraction of the digested sample’/probe fraction of the corresponding undigested sample). Experiments were performed in duplicates and averaged. Data were dichotomized in line with the MSP results. Samples with an average probe ratio >0.25 was scored as methylated. |
|  |  | 67% (2/3), MSP  50% (2/4), MS-MLPA | Anaplastic oligoastrocytoma (AOA),  WHO grade III |  |  |  |
|  |  | 75% (3/4), MSP  75% (3/4), MS-MLPA | Anaplastic astrocytoma (AA),  WHO grade III |  |  |  |
|  |  | 100% (2/2), MSP  100% (2/2), MS-MLPA | Gliosarcoma (GS),  WHO grade IV |  |  |  |
|  |  | 59% (17/29 ^k^), MSP  48% (16/33), MS-MLPA | Glioblastoma (GBM),  WHO grade IV |  |  |  |
| Kamiryo  (2004) [45] | 45% (52/116) | 45% (19/42) | Anaplastic astrocytoma (AA), primary,  WHO grade III | Frozen | MSP | Conventional MSP primers.^b^ |
|  |  | 45% (33/74) | Glioblastoma (GBM), primary,  WHO grade IV |  |  |  |
| Karayan-Tapon  (2010) [46] | 68% (55/81), MSP | - | Glioblastoma (GBM),  WHO grade IV | Frozen | MSP  Nested two-stage approach  SQ-MSP  Pyrosequencing  5 CpG sites | MSP using conventional primers for nested two-stage approach.^d^ SQ-MSP and pyrosequencing not dichotomized. |
| Koos  (2010) [47] | 46% (53/116) | - | Glioblastoma (GBM) (n=100),  WHO grade IV | FFPE | MSP | Primers as reported by Wick *et al.* (2007) [26]. |
|  |  | - | Anaplastic astrocytoma (AA) (n=16),  WHO grade III |  |  |  |

| Laffaire  (2011) [48] | 56% (23/41), qMSP  38% (18/48), Bead array^l^ | 100% (5/5), qMSP  43% (3/7), Bead array | Anaplastic astrocytoma (A) (n=1), anaplastic oligoastrocytoma (AOA) (n=2) and anaplastic oligodendroglioma (AO) (n=4),  WHO grade III | Frozen | qMSP  Bead array | qMSP using MGMT primers and probe as reported by Widschwendter (2004) [49]. β-actin was analyzed for normalization. Methylated target in each sample was calculated by dividing the MGMT/ β-actin ratio in the sample by the same ratio form a 100% methylated reference.  Bead array, samples with a β-value >0.2 were considered to be methylated. With this cut-off value, the consistency between qMSP and bead array results was 70.1 %. |
| --- | --- | --- | --- | --- | --- | --- |
|  |  | 50% (18/36), qMSP  37% (15/41), Bead array | Glioblastoma (GBM) (n=41),  WHO grade IV |  |  |  |
| Lai  (2011) [50] | 41% (29/70) | - | Glioblastoma (GBM), newly diagnosed,  WHO grade IV | FFPE | MSP  Nested two-stage | Conventional primers for nested two-stage approach.^d^ |
| Lavon  (2010) [51] | 41% (17/41) | - | High grade astrocytic tumor  WHO grade III (n=12) and IV (n=29) | FFPE | MSP | Using MGMT metylated primers and β-actin for normalization. Conventional MSP primers^b^ for the methylated MGMT reaction. |
| Levner  (2009) [52] | 53% (31/59) | - | Glioblastoma (GBM), newly diagnosed,  WHO grade IV | FFPE | MSP  Nested two-stage approach | Conventional primers for nested two-stage approach.^d^ |
| Liu  (2010) [53] | 97% (64/66) | 100% (43/43) | Glioblastoma (GBM),  primary (n=35) and secondary (n=8),  WHO grade IV | - | Real-time PCR  after immunocapturing of methylated DNA | Reporting primers. The relative enrichment of methylation in each sample was defined as the ratio of the signal in the immunoprecipitated DNA vs. input DNA. The ratio was standardized against an unmehtylated control. |
|  |  | 91% (21/23) | Anaplastic astrocytoma (A) (n=18),  anaplastic oligoastrocytoma (AOA) (n=3) and anaplastic oligodendroglioma (AO) (n=2),  WHO grade III |  |  |  |
| Lorente  (2009) [54] | 60% (29/48), MSP  58% (28/48), MCA-Meth | 60% (6/10),  in both methods | Anaplastic astrocytoma (AA),  WHO grade III | Frozen | MSP  MCA-meth. | MSP and MCA-meth primers designed by the authors, sequences available upon request. |
|  |  | 61% (23/38), MSP  58% (22/38), MCA-Meth | Glioblastoma (GBM),  WHO grade IV |  |  |  |
| Martinez  (2007) [55] | 41% (80/195) | 39% (73/186) | Glioblastoma (GBM),  survival time ≤ 1 year,  WHO grade IV | FFPE  and  Frozen | MSP | Conventional MSP primers.^b,c^ MSP performed a second time as validation with other primers (primer sequences, available upon request). |
|  |  | 78% (7/9) | Glioblastoma (GBM),  survival time >3 years,  WHO grade IV |  |  |  |
| Martinez  (2009) [56] | 11% (5/46) | - | Glioblastoma (GBM),  WHO grade IV | Frozen | Bead array | Mean methylation (β-value) in one normal brain was 0.02. Samples with a β-value >0.5 were considered to be methylated. |
| Martini  (2008) [57] | 43% (20/46) | - | Glioblastoma (GBM),  WHO grade IV | FFPE | MSP  Nested two-stage approach | Conventional primers for nested two-stage approach.^d^ |
| Maxwell  (2006) [3] | 62% (18/29) | 67% (14/21) | Glioblastoma (GBM),  WHO grade IV | Frozen in Tissue-Tek OCT compound | qMSP | Methodology devised by OncoMethylome Sciences, Inc. Calculating the MGMT/ β-actin ratios (copies methylated MGMT/ copies β-actin × 1000).  Cut offs based on comparison of the results from OncoMethylome with the gel based MSP assay used by Hegi *et al.* (2005) [40]. Samples with a result >12 were considered to be methylated. |
|  |  | 50% (4/8) | Anaplastic oligoastrocytoma (AO) (n=3) and anaplastic astrocytoma (AA), (n=5),  WHO grade III |  |  |  |
| Mellai  (2009) [58] | 29% (29/101) | - | Glioblastoma (GBM),  WHO grade IV | FFPE | MSP | Conventional MSP primers.^b^ The methylated and unmethylated reactions from the MSP were analyzed by capillary electrophoresis, and the peak height ratio (methylated/unmethylated allele was calculated. Samples was scored as methylated if the peak hight ratio was >0.1.  When including samples with a ratio between 0.01 and 0.09,(weak methylation peak, n=7), the methylation frequency increased to 35.6%. The same frequency was found when calculating the occurrence of the methylated allel only. |
| Metellus  (2009) [59] |  | 29% (6/21),  in both methods | Glioblastoma (GBM), primary tumor,  WHO grade IV | Frozen | MSP  qMSP | MSP using conventional MSP primers^b^  qMSP using primers and probes as reported by Widschwendter (2004) [49], using *Col2A1* to normalize. The PMR-value (%) was calculated and samples were considered methylated if the PMR was >0%. |
|  |  | 32% (6/19) MSP  26% (5/19) qMSP | Glioblastoma (GBM), recurrent tumor,  WHO grade IV |  |  |  |
| Mikeska  (2007) [60] | 41% (9/22), BS | - | Glioblastoma (GBM), primary,  WHO grade IV | Frozen | BS  27 CpG sites  COBRA  SIRPH  Pyrosequencing  4 CpG sites | Including 3 normal brain controls.  BS, determination of methylation status by evaluating the dendrogram for hierarchical cluster analysis of 25 CpG sites. Results obtained using COBRA, SIRPH and pyrosequencing is compared to the BS results and not dichotomized. All primers reported. |
| Mikkelsen  (2009) [61] | 54% (13/24) | - | Anaplastic oligoastrocytoma (AOA) and anaplastic oligodendroglioma (AO), newly diagnosed  WHO grade III | - | MSP | Not clear if the MSP was performed in a two-step approach. Probably using conventional MSP primers.^b, c^ |
| Minniti  (2010) [62] | 36% (31/87) | - | Glioblastoma (GBM), newly diagnosed (detection of recurrence by follow-up MRI)  WHO grade IV | FFPE | MSP | Conventional MSP primers.^b^ |
| Morandi  (2010) [63] | 43% (69/159), MSP  44% (70/159), qMSP | - | Glioblastoma (GBM), newly diagnosed  WHO grade IV | FFPE | MSP  Nested two-stage approach  qMSP | MSP using conventional primers for nested two-stage approach.^d,e^ Not specifying first stage primers.  qMSP using LNA primers. Conventional MSP primers ^b^ modified at 3’end, and beacon probes including two additional CpG sites. Methylated and unmethylated *SNURF* was analyzed as reference reactions and a mixture of 50% methylated and unmethylated DNA served as a calibrator. Samples with a normalized ratio value mMGMT/uMGMT alleles >0.001 was considered to be methylated. |
| Möllemann  (2005) [37] | 91% (31/34), MSP  94% (32/34), BS | 96% (22/23),  In both methods | Anaplastic oligodendroglioma (AO),  WHO grade III | Frozen | MSP  BS  25 CpG sites | MSP using conventional MSP primers.^b^  BS, sample was considered methylated if more than 50% of the sequenced CpG sites were metylated. |
|  |  | 82% (9/11), MSP  91% (10/11), BS | Anaplastic oligoastrocytoma (AOA),  WHO grade III |  |  |  |
| Nakamura  (2001) [64] | 48% (25/52) | 36% (13/36) | Glioblastoma (GBM), primary,  WHO grade IV | FFPE | MSP | Conventional MSP primers.^b^ |
|  |  | 75% (12/16) | Glioblastoma (GBM), secondary,  WHO grade IV |  |  |  |
| Park  (2009) [65] | 54% (26/48) | - | Glioblastoma (GBM), newly diagnosed,  WHO grade IV | FFPE | MSP | Conventional MSP primers.^b^ |
| Parrella  (2009) [66] | 69% (24/35), qMSP | - | Glioblastoma (GBM),  WHO grade IV | FFPE  and  Frozen | qMSP  MSP | qMSP, calculating the MGMT/ β-actin ratios (copies methylated MGMT/ copies β-actin × 1000).  The ratio indicates the relative level of methylated DNA in each sample. Cut-off value (>0.35) based on ROC curve analysis of tumor and normal brain tissue (n=24). Primers and probes were as reported by Hoque *et al.* (2006) [67].  MSP, using same primer sequences as for the methylated qMSP reaction. As control of the bisulphite conversion a part of the β-actin promoter region not containing CpGs was amplified. Methylation frequency determined by MSP is not reported for this specific diagnose. |

| Paz  (2004) [68] | 30% (28/92) | 30% (6/20) | Anaplastic oligodendroglioma (AO), primary,  WHO grade III | - | MSP | Conventional MSP primers.^b,c^ Performed MSP a second time with other primers (reported). The results from the two assays were identical. |
| --- | --- | --- | --- | --- | --- | --- |
|  |  | 0% (0/4) | Anaplastic oligoastrocytoma (AOA), primary,  WHO grade III |  |  |  |
|  |  | 13% (2/16) | Anaplastic astrocytoma (AA), primary,  WHO grade III |  |  |  |
|  |  | 38% (20/52) | Glioblastoma (GBM), primary,  WHO grade IV |  |  |  |
| Piccirilli  (2006) [69] | 59% (13/22) | - | Glioblastoma (GBM),  patients >80 years,  WHO grade IV | Frozen | MSP | Not reporting primers or referring. |
| Pirtoli  (2009) [70] | 56% (29/52) | - | Anaplastic astrocytoma (AA),  WHO grade III and glioblastoma (GBM),  WHO grade IV | FFPE | MSP | Not reporting primers or referring. |
| Prados  (2009) [71] | 36% (16/44) | - | Glioblastoma (GBM) and gliosarcoma (GS), newly diagnosed,  WHO grade IV | - | MSP | Conventional MSP primers.^b^ |
| Ramirez  (2003) [72] | 38% (8/21) | - | Glioblastoma (GBM),  WHO grade IV | Frozen | MSP | Conventional MSP primers.^b^ |
| Rivera  (2010) [73] | 24% (54/225) | - | Glioblastoma (GBM), newly diagnosed,  WHO grade IV | FFPE | qMSP | qMSP using primers and probes as reported by Widschwendter (2004) [49]. Included *Col2A1* as a control. Samples with amplification of methylated sequence, or both methylated and unmethylated sequences, were interpereted as methylated. This corresponded to a cut off of ≥ 0.05 fluorescence of FAM after at least 40 PCR cycles. |
| Rodriguez  (2008) [74] | 38% (15/39) | - | Glioblastoma (GBM),  WHO grade IV | FFPE | MSP | Conventional MSP primers^b^, labelled with fluorescens. |
| Sadones  (2009) [75] | 26% (10/38) | - | Anaplastic astrocytoma (AA) (n=12) and anaplastic oligoastrocytoma (AOA) (n=4), recurrent, WHO grade III  Glioblastoma (GBM) (n=22), recurrent,  WHO grade IV | FFPE | qMSP | OncoMethylome Sciences, Inc. Calculating the MGMT/ β-actin ratios (copies methylated MGMT/ copies β-actin × 1000). Samples with a result >12 were considered to be methylated. |
| Schaich  (2009) [76] | 34% (37/110) | - | Glioblastoma (GBM),  WHO grade IV | Frozen | MSP | Primers as reported by Wick *et al.* (2007) [26]. |
| Shamsara  (2009) [77] | 48% (24/50) | - | Glioblastoma (GBM), primary,  WHO grade IV | FFPE | MSP | Conventional MSP primers.^b^ |
| Sijben  (2008) [78] | 45% (13/29) | - | Glioblastoma (GBM), newly diagnosed, patients ≥ 65 years,  WHO grade IV | FFPE | MSP  Nested two-stage approach | Conventional primers for nested two-stage approach.^d^ |
| Slaby  (2010) [79] | 45% (10/22) | - | Glioblastoma (GBM), primary,  WHO grade IV | FFPE | qMSP | Conventional MSP primer sequences.^b^ Primers amplifying unconverted genomic DNA for β-actin were used as an internal standard to assess bisulfite conversion efficiency. |
| Smith  (2008) [80] | 52% (12/23) | - | Glioblastoma (GBM), newly diagnosed,  WHO grade IV | FFPE | MSP  Nested two-stage approach | Reporting primers. |
| Sonoda  (2009) [81] | 50% (15/30) | 79% (11/14) | Glioblastoma (GBM), newly diagnosed,  survival >3 years,  WHO grade IV | Frozen | MSP | Conventional MSP primers.^b^ |
|  |  | 25% (4/16) | Glioblastoma (GBM), newly diagnosed,  survival <1.5 years,  WHO grade IV |  |  |  |
| Sonoda  (2010) [82] | 56% (35/62) | - | Glioblastoma (GBM), newly diagnosed,  WHO grade IV | Frozen | MSP | Conventional MSP primers.^b^ |
| Spiegl-Kreinecker  (2010) [83] | 66% (47/71) | - | Glioblastoma (GBM) (n = 69) and  gliosarcoma (GS) (n=2), primary,  WHO grade IV | Frozen  and  Cell explants | MSP | Conventional MSP primers.^b^  Cell cultures were established from surgery specimens, and analyzed between passages 2 and 6. |
| Stupp  (2010) [84] | 51% (23/45) | - | Glioblastoma (GBM), newly diagnosed  WHO grade IV | FFPE  and Cryosections | MSP  Nested two-stage approach | Conventional primers for nested two-stage approach.^d^ |
| van den Bent  (2009) [85] | 80% (110/137) | 84% (81/97) | Anaplastic oligodendroglioma (AO) and anaplastic oligoastrocytoma (AOA) without necrosis,  WHO grade III | FFPE | MS-MLPA | MS-MLPA results were normalized by calculating the peak height ratio of MGMT probe signal relative to the control fragments. Further the normalized values of the MGMT probes within digested DNA samples were divided by normalized values of corresponding undigested samples, to evaluate the degree of methylation. These ratios were averaged for each probe. Results >0.25 indicated methylation. Cut off determined by the manufacturer |
|  |  | 73% (29/40) | Glioblastoma (GBM),  WHO grade IV |  |  |  |
| Vogelbaum  (2008) [86] | 80% 16/20 | - | Anaplastic oligodendroglioma (AO) and anaplastic oligoastrocytoma (AOA), newly diagnosed,  WHO grade III | - | MSP | Conventional MSP primers.^b^ |
| Watanabe  (2005) [87] | 38% (17/45) | 44% (7/16) | Anaplastic astrocytoma (AA), newly diagnosed,  WHO grade III | FFPE | MSP | Conventional MSP primers.^b^ |
|  |  | 34% (10/29) | Glioblastoma (GBM), newly diagnosed,  WHO grade IV |  |  |  |
| Watanabe  (2006) [88] | 35% (7/20) | 56% (5/9) | Anaplastic astrocytoma (AA),  WHO grade III | Frozen | MSP | Conventional MSP primers.^b^ |
|  |  | 18% (2/11) | Glioblastoma (GBM),  WHO grade IV |  |  |  |
| Weiler  (2010) [89] | 41% (16/39) | - | Glioblastoma (GBM), newly diagnosed,  WHO grade IV | FFPE | MSP | Conventional MSP primers.^b^ |
| Weller  (2009) [90] | 44% (133/295) | - | Glioblastoma (GBM), newly diagnosed,  WHO grade IV | Frozen | MSP | Primers as reported by Wick *et al.* (2007) [26]. |
| Wemmert  (2009) [91] | 56% (15/27) | 48% (11/23) | Glioblastoma (GBM), primary,  WHO grade IV | Frozen | MSP | Primers as reported by Paz *et al.* (2004) [68]. Not specifying if it is the conventional primers^b^ or the other reported primers set. |
|  |  | 100% (4/4 ) | Glioblastoma (GBM), secondary,  WHO grade IV |  |  |  |
| Wick  (2007) [26] | 47% (17/36) | - | Glioblastoma (GBM), recurrent  WHO grade IV | FFPE | MSP | Primers as reported by Wick *et al.* (2007) [26]. |
| Wick  (2009) [92] | 61% (123/202) | 50% (48/96) | Anaplastic astrocytoma (AA),  WHO grade III | FFPE | MSP | Conventional MSP primers^b^, or primers as reported by Wick *et al.* (2007) [26]. Not stated clearly. |
|  |  | 71% (53/75) | Anaplastic oligoastrocytoma (AOA),  WHO grade III |  |  |  |
|  |  | 71% (22/31) | Anaplastic oligodendroglioma (AO),  WHO grade III |  |  |  |
| Yachi  (2008) [93] | 32% (8/25 ^m^), MSP  38% (8/21), BS | - | Glioblastoma (GBM), newly diagnosed,  WHO grade IV | Frozen | MSP  BS  27 CpG sites | MSP using conventional MSP primers.^b^  BS using primers as reported by Mikeska *et al.* (2007) [60]. A CpG site was considered methylated If the cytosine and thymine peaks was equally sized or the cytosine peak being larger. (Based on the criteria described by Grasbon-Frodl *et al.* (2007)). |
| Yang  (2009) [94] | 89% (32/36) | 100% (19/19) | Anaplastic oligodendroglioma (AO), newly diagnosed,  WHO grade III | FFPE | MSP | Conventional MSP primers.^b^ |
|  |  | 100% (7/7) | Anaplastic oligoastrocytoma (AOA), newly diagnosed,  WHO grade III |  |  |  |
|  |  | 60% (6/10) | Anaplastic astrocytoma (AA),  newly diagnosed,  WHO grade III |  |  |  |
| Zawlik  (2009) [95] | 44% (165/371) | 43% (149/349) | Glioblastoma (GBM), primary,  WHO grade IV | FFPE | MSP  Nested two stage approach | Conventional primers for nested two-stage approach.^d^ |
|  |  | 73% (16/22) | Glioblastoma (GBM), secondary,  WHO grade IV |  |  |  |

1. When possible to deduce from the publication (directly or from supplementary material).
2. Conventional MSP primers, as reported by Esteller *et al.* (1999) [1].
3. A possible spelling error in reported primers, overall quite similar to the conventional primers.
4. Conventional primers for nested two-stage approach: first step PCR primers, as reported by Palmisano *et al.* (2000) [96]. Conventional MSP primers^b^ are used in the second step.
5. Optional first step primers used in MSP (nested two-stage approach), as reported by van Engeland *et al.* (2003) [97].
6. Reference missing in publication. Most likely optional first step primers as reported by van Engeland *et al.* (2003) [97].
7. 9 patients included in the pre-treatment group.
8. Most likely qMSP.
9. Reporting methylation frequencies for samples included in the tumor texture analysis (53%) and tumor location analysis (50%). Most patients are included in both analyses.
10. We considered the promoter to be methylated if the amplicon from methylated primers could be seen alone or if both the methylated and unmethylated amplicon was present.
11. Originally 31 glioblastoma samples were analyzed by MSP, but two of them are excluded here, as they did not produce PCR-products with methylated nor unmethylated primers.
12. Study includes 8 patients which are represented with two tumor samples, initial and recurrent.
13. Methylation status in 9 samples was previously reported by Watanabe *et al.* (2006) [88].

Abbreviations: MSP, methylation specific PCR; FFPE, formalin-fixed paraffin-embedded; qMSP, quantitative methylation specific PCR; MS-HMR, methylation sensitive high resolution melting; MALDI-TOF MS, Matrix-assisted laser desorption/ionisation-time of flight mass spectrometry; SQ-MSP, semiquantitative methyl-speciﬁc polymerase chain reaction; MCA-Meth, Melting curve analysis-based real-time methylation assay; PMR, percentage of methylated reference; COBRA, combined bisulphite restriction analysis; SIRPH, SNuPE ion pair-reverse phase high-performance liquid chromatography); ROC, Receiver Operating Characteristics; MS-MLPA, methylation speciﬁc-multiplex ligation-dependent probe ampliﬁcation; BS, Bisulfite sequencing; OCT, optimum cutting temperature; LNA, locked nucleic acid

| SUPPLEMENTARY TABLE 2: Studies of *MGMT* promoter methylation in low-grade gliomas - summary of methylation frequencies and methodological details. | | | | | | |
| --- | --- | --- | --- | --- | --- | --- |
| **Low-grade glioma studies** | | | | | | |
| **First author (year)** | **Total methylation frequency %** | **Subgroup**  **methylation frequency^a^** | **Diagnosis** | **Tissue** | **Method** | **Methodological details** |
| Alonso  (2003) [98] | 77% (17/22) | - | Oligodendroglioma (O),  WHO grade II | Frozen | MSP | Conventional MSP primers.^b^ |
| Cankovic  (2007) [15] | 28% (11/40) | 33% (2/6) | Astrocytoma (A),  WHO grade II | FFPE | MSP  two-step approach | Conventional MSP primers.^b,c^ |
|  |  | 26% (8/31) | Oligodendroglioma (O),  WHO grade II |  |  |  |
|  |  | 33% (1/3) | Oligodendroglioma (O) + astrocytoma (A),  WHO grade II |  |  |  |
| Dong  (2001) [99] | 57% (16/28) | 53%(10/19) | Oligodendroglioma (O),  WHO grade II | - | MSP | Conventional MSP primers.^b^ |
|  |  | 67% (6/9) | Oligoastrocytoma (OA),  WHO grade II |  |  |  |
| Esteller  (1999) [1] | 31% (8/26) | - | Astrocytoma (A) and oligodendroglioma (O), WHO grade II | - | MSP | Conventional MSP primers.^b^ |
| Everhard  (2006) [100] | 93% (63/68) | 93% (39/42) | Oligodendroglioma (O),  WHO grade II | Frozen | MSP  Nested  two-stage approach | Conventional primers for nested two-stage approach.^d^ |
|  |  | 94% (17/18) | Oligoastrocytoma (OA),  WHO grade II |  |  |  |
|  |  | 88% (7/8) | Astrocytoma (A),  WHO grade II |  |  |  |
| Gonzalez-Gomez  (2003) [35] | 50% (12/24) | - | Astrocytoma (A),  WHO grade II | Frozen | MSP | Conventional MSP primers.^b^ |
| Houillier  (2010) [101] | 81% (150/185) | - | Astrocytoma (A), oligodendroglioma (O) and oligoastrocytoma (OA), WHO grade II | - | MSP  Nested  two-stage approach | Conventional primers for nested two-stage approach.^d^ |
| Kesari  (2009) [102] | 60% (12/20) | - | Oligodendroglioma (O), astrocytoma (A) and oligoastrocytomas (OA), WHO grade II | FFPE | e | OncoMethylome Sciences, Inc. |
| Komine  (2003) [103] | 43% (21/49) | - | Fibrillary astrocytoma (A) newly diagnosed,  WHO grade II | FFPE | MSP | Conventional MSP primers.^b^ |
| Kuo  (2009) [104] | 42% (15/36) | 34% (10/29) | Oligodendroglioma (O),  WHO grade II | FFPE | MSP | Conventional MSP primers.^b^ |
|  |  | 71% (5/7) | Oligoastrocytoma (OA),  WHO grade II |  |  |  |
| Laffaire  (2011) [48] | 80% (24/30), qMSP  37% (14/38), Bead array^f^ | 80% (4/5), qMSP  60% (3/5),  Bead array | Astrocytoma (A),  WHO grade II | Frozen | qMSP  Bead array | qMSP using MGMT primers and probe as reported by Widschwendter (2004)[49]. β-actin was analyzed for normalization. Methylated target in each sample was calculated by dividing the MGMT/ β-actin ratio in the sample by the same ratio form a 100% methylated reference.  Bead array, samples with a β-value >0.2 were considered to be methylated. With this cut-off value, the consistency between qMSP and bead array results was 70.1 %. |
|  |  | 78% (7/9), qMSP  56% (9/16), Bead array | Oligodendroglioma (O),  WHO grade II |  |  |  |
|  |  | 81% (13/16), qMSP  12% (2/17), Bead array | Oligoastrocytoma (OA),  WHO grade II |  |  |  |
| Nakamura  (2001) [64] | 48% (26/54) | - | Astrocytoma (A),  WHO grade II | FFPE | MSP | Conventional MSP primers.^b^ |
| Tosoni  (2008) [105] | 47% (14/30) | 50% (9/18) | Oligodendroglioma (O),  WHO grade II | FFPE | MSP  Nested two-stage approach | Conventional primers for nested two-stage approach^d^, some cases amplified with optional first stage primers.^g^ |
|  |  | 42% (5/12) | Astrocytoma (A) and oligoastrocytoma (OA), WHO grade II |  |  |  |
| Watanabe  (2002) [106] | 48% (20/42) | 47% (9/19) | Oligodendroglioma (O),  WHO grade II | FFPE | MSP | Conventional MSP primers.^b^ |
|  |  | 48% (11/23)^h^ | Fibrillary astrocytoma (A),  WHO grade II |  |  |  |

1. When possible to deduce from the publication (directly or from supplementary material).
2. Conventional MSP primers, as reported by Esteller *et al.* (1999) [1].
3. A possible spelling error in reported primers, overall quite similar to the conventional primers.
4. Conventional primers for nested two-stage approach: first step PCR primers, as reported by Palmisano *et al.* (2000) [96]. Conventional MSP primers^b^ are used in the second stage.
5. Most likely quantitative MSP (qMSP).
6. Study includes 8 patients which are represented with two tumor samples, initial and recurrent.
7. Optional first stage primers used in MSP (nested two-stage approach), as reported by van Engeland *et al.* (2003) [97].
8. MGMT status for 12 of these samples has been reported previously by Nakamura *et al.* (2001) [64].

Abbreviations: MSP, methylation specific PCR; FFPE, formalin-fixed paraffin-embedded; qMSP, quantitative methylation specific PCR

**References to Supplementary tables 1 and 2:**

1. Esteller M, Hamilton SR, Burger PC, Baylin SB, Herman JG: **Inactivation of the DNA repair gene O6-methylguanine-DNA methyltransferase by promoter hypermethylation is a common event in primary human neoplasia.** *Cancer Res* 1999, **59:**793-797.

2. Everhard S, Tost J, El Abdalaoui H, Criniere E, Busato F, Marie Y, Gut IG, Sanson M, Mokhtari K, Laigle-Donadey F, Hoang-Xuan K, Delattre JY, Thillet J: **Identification of regions correlating MGMT promoter methylation and gene expression in glioblastomas.** *Neuro Oncol* 2009, **11:**348-356.

3. Maxwell JA, Johnson SP, Quinn JA, McLendon RE, Ali-Osman F, Friedman AH, Herndon JE, 2nd, Bierau K, Bigley J, Bigner DD, Friedman HS: **Quantitative analysis of O6-alkylguanine-DNA alkyltransferase in malignant glioma.** *Mol Cancer Ther* 2006, **5:**2531-2539.

4. Ang C, Guiot M-C, Ramanakumar AV, Roberge D, Kavan P: **Clinical significance of molecular biomarkers in glioblastoma.** *Can J Neurol Sci* 2010, **37:**625-630.

5. Balaña C, Ramirez JL, Taron M, Roussos Y, Ariza A, Ballester R, Sarries C, Mendez P, Sanchez JJ, Rosell R: **O6-methyl-guanine-DNA methyltransferase methylation in serum and tumor DNA predicts response to 1,3-bis(2-chloroethyl)-1-nitrosourea but not to temozolamide plus cisplatin in glioblastoma multiforme.** *Clin Cancer Res* 2003, **9:**1461-1468.

6. Blanc JL, Wager M, Guilhot J, Kusy S, Bataille B, Chantereau T, Lapierre F, Larsen CJ, Karayan-Tapon L: **Correlation of clinical features and methylation status of MGMT gene promoter in glioblastomas.** *J Neurooncol* 2004, **68:**275-283.

7. Brandes AA, Tosoni A, Cavallo G, Bertorelle R, Gioia V, Franceschi E, Biscuola M, Blatt V, Crino L, Ermani M: **Temozolomide 3 weeks on and 1 week off as first-line therapy for recurrent glioblastoma: phase II study from gruppo italiano cooperativo di neuro-oncologia (GICNO).** *Br J Cancer* 2006, **95:**1155-1160.

8. Brandes AA, Tosoni A, Cavallo G, Reni M, Franceschi E, Bonaldi L, Bertorelle R, Gardiman M, Ghimenton C, Iuzzolino P, Pession A, Blatt V, Ermani M: **Correlations between O6-methylguanine DNA methyltransferase promoter methylation status, 1p and 19q deletions, and response to temozolomide in anaplastic and recurrent oligodendroglioma: a prospective GICNO study.** *J Clin Oncol* 2006, **24:**4746-4753.

9. Brandes AA, Franceschi E, Tosoni A, Blatt V, Pession A, Tallini G, Bertorelle R, Bartolini S, Calbucci F, Andreoli A, Frezza G, Leonardi M, Spagnolli F, Ermani M: **MGMT promoter methylation status can predict the incidence and outcome of pseudoprogression after concomitant radiochemotherapy in newly diagnosed glioblastoma patients.** *J Clin Oncol* 2008, **26:**2192-2197.

10. Brandes AA, Tosoni A, Franceschi E, Sotti G, Frezza G, Amista P, Morandi L, Spagnolli F, Ermani M: **Recurrence pattern after temozolomide concomitant with and adjuvant to radiotherapy in newly diagnosed patients with glioblastoma: correlation With MGMT promoter methylation status.** *J Clin Oncol* 2009, **27:**1275-1279.

11. Brandes AA, Franceschi E, Tosoni A, Benevento F, Scopece L, Mazzocchi V, Bacci A, Agati R, Calbucci F, Ermani M: **Temozolomide concomitant and adjuvant to radiotherapy in elderly patients with glioblastoma: correlation with MGMT promoter methylation status.** *Cancer* 2009, **115:**3512-3518.

12. Brandes AA, Franceschi E, Tosoni A, Bartolini S, Bacci A, Agati R, Ghimenton C, Turazzi S, Talacchi A, Skrap M, Marucci G, Volpin L, Morandi L, Pizzolitto S, Gardiman M, Andreoli A, Calbucci F, Ermani M: **O(6)-methylguanine DNA-methyltransferase methylation status can change between first surgery for newly diagnosed glioblastoma and second surgery for recurrence: clinical implications.** *Neuro Oncol* 2010, **12:**283-288.

13. Brell M, Tortosa A, Verger E, Gil JM, Vinolas N, Villa S, Acebes JJ, Caral L, Pujol T, Ferrer I, Ribalta T, Graus F: **Prognostic significance of O6-methylguanine-DNA methyltransferase determined by promoter hypermethylation and immunohistochemical expression in anaplastic gliomas.** *Clin Cancer Res* 2005, **11:**5167-5174.

14. **Comprehensive genomic characterization defines human glioblastoma genes and core pathways. The Cancer Genome Atlas Research Network.** *Nature* 2008, **455:**1061-1068.

15. Cankovic M, Mikkelsen T, Rosenblum ML, Zarbo RJ: **A simplified laboratory validated assay for MGMT promoter hypermethylation analysis of glioma specimens from formalin-fixed paraffin-embedded tissue.** *Lab Invest* 2007, **87:**392-397.

16. Cao VT, Jung TY, Jung S, Jin SG, Moon KS, Kim IY, Kang SS, Park CS, Lee KH, Chae HJ: **The correlation and prognostic significance of MGMT promoter methylation and MGMT protein in glioblastomas.** *Neurosurgery* 2009, **65:**866-875; discussion 875.

17. Christmann M, Nagel G, Horn S, Krahn U, Wiewrodt D, Sommer C, Kaina B: **MGMT activity, promoter methylation and immunohistochemistry of pretreatment and recurrent malignant gliomas: a comparative study on astrocytoma and glioblastoma.** *Int J Cancer* 2010, **127:**2106-2118.

18. Clarke JL, Iwamoto FM, Sul J, Panageas K, Lassman AB, DeAngelis LM, Hormigo A, Nolan CP, Gavrilovic I, Karimi S, Abrey LE: **Randomized phase II trial of chemoradiotherapy followed by either dose-dense or metronomic temozolomide for newly diagnosed glioblastoma.** *J Clin Oncol* 2009, **27:**3861-3867.

19. Costa BM, Smith JS, Chen Y, Chen J, Phillips HS, Aldape KD, Zardo G, Nigro J, James CD, Fridlyand J, Reis RM, Costello JF: **Reversing HOXA9 oncogene activation by PI3K inhibition: epigenetic mechanism and prognostic significance in human glioblastoma.** *Cancer Res* 2010, **70:**453-462.

20. Costa BM, Caeiro C, Guimarães I, Martinho O, Jaraquemada T, Augusto I, Castro L, Osorio L, Linhares P, Honavar M, Resende M, Braga F, Silva A, Pardal F, Amorim J, Nabico R, Almeida R, Alegria C, Pires M, Pinheiro C, Carvalho E, Lopes JM, Costa P, Damasceno M, Reis RM: **Prognostic value of MGMT promoter methylation in glioblastoma patients treated with temozolomide-based chemoradiation: a Portuguese multicentre study.** *Oncol Rep* 2010, **23:**1655-1662.

21. Crinière E, Kaloshi G, Laigle-Donadey F, Lejeune J, Auger N, Benouaich-Amiel A, Everhard S, Mokhtari K, Polivka M, Delattre JY, Hoang-Xuan K, Thillet J, Sanson M: **MGMT prognostic impact on glioblastoma is dependent on therapeutic modalities.** *J Neurooncol* 2007, **83:**173-179.

22. Drabycz S, Roldán G, de Robles P, Adler D, McIntyre JB, Magliocco AM, Cairncross JG, Mitchell JR: **An analysis of image texture, tumor location, and MGMT promoter methylation in glioblastoma using magnetic resonance imaging.** *Neuroimage* 2010, **49:**1398-1405.

23. Ducray F, de Reyniès A, Chinot O, Idbaih A, Figarella-Branger D, Colin C, Karayan-Tapon L, Chneiweiss H, Wager M, Vallette F, Marie Y, Rickman D, Thomas E, Delattre JY, Honnorat J, Sanson M, Berger F: **An ANOCEF genomic and transcriptomic microarray study of the response to radiotherapy or to alkylating first-line chemotherapy in glioblastoma patients.** *Mol Cancer* 2010, **9:**234.

24. Dunn J, Baborie A, Alam F, Joyce K, Moxham M, Sibson R, Crooks D, Husband D, Shenoy A, Brodbelt A, Wong H, Liloglou T, Haylock B, Walker C: **Extent of MGMT promoter methylation correlates with outcome in glioblastomas given temozolomide and radiotherapy.** *Br J Cancer* 2009, **101:**124-131.

25. El Hindy N, Adamzik M, Lambertz N, Bachmann HS, Worm K, Egensperger R, Frey UH, Asgari S, Sure U, Siffert W, Sandalcioglu IE: **Association of the GNB3 825T-allele with better survival in patients with glioblastoma multiforme.** *J Cancer Res Clin Oncol* 2010, **136:**1423-1429.

26. Wick A, Felsberg J, Steinbach JP, Herrlinger U, Platten M, Blaschke B, Meyermann R, Reifenberger G, Weller M, Wick W: **Efficacy and tolerability of temozolomide in an alternating weekly regimen in patients with recurrent glioma.** *J Clin Oncol* 2007, **25:**3357-3361.

27. Eoli M, Menghi F, Bruzzone MG, De Simone T, Valletta L, Pollo B, Bissola L, Silvani A, Bianchessi D, D'Incerti L, Filippini G, Broggi G, Boiardi A, Finocchiaro G: **Methylation of O6-methylguanine DNA methyltransferase and loss of heterozygosity on 19q and/or 17p are overlapping features of secondary glioblastomas with prolonged survival.** *Clin Cancer Res* 2007, **13:**2606-2613.

28. Esteller M, Garcia-Foncillas J, Andion E, Goodman SN, Hidalgo OF, Vanaclocha V, Baylin SB, Herman JG: **Inactivation of the DNA-repair gene MGMT and the clinical response of gliomas to alkylating agents.** *N Engl J Med* 2000, **343:**1350-1354.

29. Etcheverry A, Aubry M, de Tayrac M, Vauleon E, Boniface R, Guenot F, Saikali S, Hamlat A, Riffaud L, Menei P, Quillien V, Mosser J: **DNA methylation in glioblastoma: impact on gene expression and clinical outcome.** *BMC Genomics* 2010, **11:**701.

30. Fabi A, Metro G, Vidiri A, Lanzetta G, Carosi M, Telera S, Maschio M, Russillo M, Sperduti I, Carapella CM, Cognetti F, Pace A: **Low-dose fotemustine for recurrent malignant glioma: a multicenter phase II study.** *J Neurooncol* 2010, **100:**209-215.

31. Felsberg J, Rapp M, Loeser S, Fimmers R, Stummer W, Goeppert M, Steiger HJ, Friedensdorf B, Reifenberger G, Sabel MC: **Prognostic significance of molecular markers and extent of resection in primary glioblastoma patients.** *Clin Cancer Res* 2009, **15:**6683-6693.

32. Gan HK, Rosenthal MA, Dowling A, Kalnins R, Algar E, Wong N, Benson A, Woods AM, Cher L: **A phase II trial of primary temozolomide in patients with grade III oligodendroglial brain tumors.** *Neuro Oncol* 2010, **12:**500-507.

33. Gerstner ER, Yip S, Wang DL, Louis DN, Iafrate AJ, Batchelor TT: **MGMT methylation is a prognostic biomarker in elderly patients with newly diagnosed glioblastoma.** *Neurology* 2009, **73:**1509-1510.

34. Glas M, Happold C, Rieger J, Wiewrodt D, Bahr O, Steinbach JP, Wick W, Kortmann RD, Reifenberger G, Weller M, Herrlinger U: **Long-term survival of patients with glioblastoma treated with radiotherapy and lomustine plus temozolomide.** *J Clin Oncol* 2009, **27:**1257-1261.

35. Gonzalez-Gomez P, Bello MJ, Arjona D, Lomas J, Alonso ME, De Campos JM, Vaquero J, Isla A, Gutierrez M, Rey JA: **Promoter hypermethylation of multiple genes in astrocytic gliomas.** *Int J Oncol* 2003, **22:**601-608.

36. Grasbon-Frodl EM, Kreth FW, Ruiter M, Schnell O, Bise K, Felsberg J, Reifenberger G, Tonn JC, Kretzschmar HA: **Intratumoral homogeneity of MGMT promoter hypermethylation as demonstrated in serial stereotactic specimens from anaplastic astrocytomas and glioblastomas.** *Int J Cancer* 2007, **121:**2458-2464.

37. Möllemann M, Wolter M, Felsberg J, Collins VP, Reifenberger G: **Frequent promoter hypermethylation and low expression of the MGMT gene in oligodendroglial tumors.** *Int J Cancer* 2005, **113:**379-385.

38. Grossman SA, Ye X, Chamberlain M, Mikkelsen T, Batchelor T, Desideri S, Piantadosi S, Fisher J, Fine HA: **Talampanel with standard radiation and temozolomide in patients with newly diagnosed glioblastoma: a multicenter phase II trial.** *J Clin Oncol* 2009, **27:**4155-4161.

39. Hegi ME, Diserens A-C, Godard S, Dietrich P-Y, Regli L, Ostermann S, Otten P, Van Melle G, de Tribolet N, Stupp R: **Clinical trial substantiates the predictive value of O-6-methylguanine-DNA methyltransferase promoter methylation in glioblastoma patients treated with temozolomide.** *Clin Cancer Res* 2004, **10:**1871-1874.

40. Hegi ME, Diserens A-C, Gorlia T, Hamou M-F, de Tribolet N, Weller M, Kros JM, Hainfellner JA, Mason W, Mariani L, Bromberg JE, Hau P, Mirimanoff RO, Cairncross JG, Janzer RC, Stupp R: **MGMT gene silencing and benefit from temozolomide in glioblastoma.** *N Engl J Med* 2005, **352:**997-1003.

41. Hervouet E, Debien E, Campion L, Charbord J, Menanteau J, Vallette FM, Cartron PF: **Folate supplementation limits the aggressiveness of glioma via the remethylation of DNA repeats element and genes governing apoptosis and proliferation.** *Clin Cancer Res* 2009, **15:**3519-3529.

42. Ishii D, Natsume A, Wakabayashi T, Hatano H, Asano Y, Takeuchi H, Shimato S, Ito M, Fujii M, Yoshida J: **Efficacy of temozolomide is correlated with 1p loss and methylation of the deoxyribonucleic acid repair gene MGMT in malignant gliomas.** *Neurol Med Chir (Tokyo)* 2007, **47:**341-349.

43. Jesien-Lewandowicz E, Jesionek-Kupnicka D, Zawlik I, Szybka M, Kulczycka-Wojdala D, Rieske P, Sieruta M, Jaskolski D, Och W, Skowronski W, Sikorska B, Potemski P, Papierz W, Liberski PP, Kordek R: **High incidence of MGMT promoter methylation in primary glioblastomas without correlation with TP53 gene mutations.** *Cancer Genet Cytogenet* 2009, **188:**77-82.

44. Jeuken JWM, Cornelissen SJB, Vriezen M, Dekkers MMG, Errami A, Sijben A, Boots-Sprenger SHE, Wesseling P: **MS-MLPA: an attractive alternative laboratory assay for robust, reliable, and semiquantitative detection of MGMT promoter hypermethylation in gliomas.** *Lab Invest* 2007, **87:**1055-1065.

45. Kamiryo T, Tada K, Shiraishi S, Shinojima N, Kochi M, Ushio Y: **Correlation between promoter hypermethylation of the O6-methylguanine-deoxyribonucleic acid methyltransferase gene and prognosis in patients with high-grade astrocytic tumors treated with surgery, radiotherapy, and 1-(4-amino-2-methyl-5-pyrimidinyl)methyl-3-(2-chloroethyl)-3-nitrosourea-based chemotherapy.** *Neurosurgery* 2004, **54:**349-357.

46. Karayan-Tapon L, Quillien V, Guilhot J, Wager M, Fromont G, Saikali S, Etcheverry A, Hamlat A, Loussouarn D, Campion L, Campone M, Vallette FM, Gratas-Rabbia-Re C: **Prognostic value of O(6)-methylguanine-DNA methyltransferase status in glioblastoma patients, assessed by five different methods.** *J Neurooncol* 2009.

47. Koos B, Peetz-Dienhart S, Riesmeier B, Fruhwald MC, Hasselblatt M: **O(6)-methylguanine-DNA methyltransferase (MGMT) promoter methylation is significantly less frequent in ependymal tumours as compared to malignant astrocytic gliomas.** *Neuropathol Appl Neurobiol* 2010, **36:**356-358.

48. Laffaire J, Everhard S, Idbaih A, Criniere E, Marie Y, de Reynies A, Schiappa R, Mokhtari K, Hoang-Xuan K, Sanson M, Delattre JY, Thillet J, Ducray F: **Methylation profiling identifies 2 groups of gliomas according to their tumorigenesis.** *Neuro Oncol* 2011, **13:**84-98.

49. Widschwendter M, Siegmund KD, Müller HM, Fiegl H, Marth C, Muller-Holzner E, Jones PA, Laird PW: **Association of breast cancer DNA methylation profiles with hormone receptor status and response to tamoxifen.** *Cancer Res* 2004, **64:**3807-3813.

50. Lai A, Tran A, Nghiemphu PL, Pope WB, Solis OE, Selch M, Filka E, Yong WH, Mischel PS, Liau LM, Phuphanich S, Black K, Peak S, Green RM, Spier CE, Kolevska T, Polikoff J, Fehrenbacher L, Elashoff R, Cloughesy T: **Phase II study of bevacizumab plus temozolomide during and after radiation therapy for patients with newly diagnosed glioblastoma multiforme.** *J Clin Oncol* 2011, **29:**142-148.

51. Lavon I, Refael M, Zelikovitch B, Shalom E, Siegal T: **Serum DNA can define tumor-specific genetic and epigenetic markers in gliomas of various grades.** *Neuro Oncol* 2010, **12:**173-180.

52. Levner I, Drabycz S, Roldan G, De Robles P, Cairncross JG, Mitchell R: **Predicting MGMT methylation status of glioblastomas from MRI texture.** *Med Image Comput Comput Assist Interv* 2009, **12:**522-530.

53. Liu B-L, Cheng J-X, Zhang W, Zhang X, Wang R, Lin H, Huo J-L, Cheng H: **Quantitative detection of multiple gene promoter hypermethylation in tumor tissue, serum, and cerebrospinal fluid predicts prognosis of malignant gliomas.** *Neuro Oncol* 2010, **12:**540-548.

54. Lorente A, Mueller W, Urdangarín E, Lazcoz P, Lass U, von Deimling A, Castresana JS: **RASSF1A, BLU, NORE1A, PTEN and MGMT expression and promoter methylation in gliomas and glioma cell lines and evidence of deregulated expression of de novo DNMTs.** *Brain Pathol* 2009, **19:**279-292.

55. Martinez R, Schackert G, Yaya-Tur R, Rojas-Marcos I, Herman JG, Esteller M: **Frequent hypermethylation of the DNA repair gene MGMT in long-term survivors of glioblastoma multiforme.** *J Neurooncol* 2007, **83:**91-93.

56. Martinez R, Martin-Subero JI, Rohde V, Kirsch M, Alaminos M, Fernandez AF, Ropero S, Schackert G, Esteller M: **A microarray-based DNA methylation study of glioblastoma multiforme.** *Epigenetics* 2009, **4:**255-264.

57. Martini M, Pallini R, Luongo G, Cenci T, Lucantoni C, Larocca LM: **Prognostic relevance of SOCS3 hypermethylation in patients with glioblastoma multiforme.** *Int J Cancer* 2008, **123:**2955-2960.

58. Mellai M, Caldera V, Annovazzi L, Chio A, Lanotte M, Cassoni P, Finocchiaro G, Schiffer D: **MGMT promoter hypermethylation in a series of 104 glioblastomas.** *Cancer Genomics Proteomics* 2009, **6:**219-227.

59. Metellus P, Coulibaly B, Nanni I, Fina F, Eudes N, Giorgi R, Barrie M, Chinot O, Fuentes S, Dufour H, Ouafik L, Figarella-Branger D: **Prognostic impact of O6-methylguanine-DNA methyltransferase silencing in patients with recurrent glioblastoma multiforme who undergo surgery and carmustine wafer implantation: a prospective patient cohort.** *Cancer* 2009, **115:**4783-4794.

60. Mikeska T, Bock C, El-Maarri O, Hubner A, Ehrentraut D, Schramm J, Felsberg J, Kahl P, Buttner R, Pietsch T, Waha A: **Optimization of quantitative MGMT promoter methylation analysis using pyrosequencing and combined bisulfite restriction analysis.** *J Mol Diagn* 2007, **9:**368-381.

61. Mikkelsen T, Doyle T, Anderson J, Margolis J, Paleologos N, Gutierrez J, Croteau D, Hasselbach L, Avedissian R, Schultz L: **Temozolomide single-agent chemotherapy for newly diagnosed anaplastic oligodendroglioma.** *J Neurooncol* 2009, **92:**57-63.

62. Minniti G, Amelio D, Amichetti M, Salvati M, Muni R, Bozzao A, Lanzetta G, Scarpino S, Arcella A, Enrici RM: **Patterns of failure and comparison of different target volume delineations in patients with glioblastoma treated with conformal radiotherapy plus concomitant and adjuvant temozolomide.** *Radiother Oncol* 2010, **97:**377-381.

63. Morandi L, Franceschi E, de Biase D, Marucci G, Tosoni A, Ermani M, Pession A, Tallini G, Brandes A: **Promoter methylation analysis of O6-methylguanine-DNA methyltransferase in glioblastoma: detection by locked nucleic acid based quantitative PCR using an imprinted gene (SNURF) as a reference.** *BMC Cancer* 2010, **10:**48.

64. Nakamura M, Watanabe T, Yonekawa Y, Kleihues P, Ohgaki H: **Promoter methylation of the DNA repair gene MGMT in astrocytomas is frequently associated with G:C --> A:T mutations of the TP53 tumor suppressor gene.** *Carcinogenesis* 2001, **22:**1715-1719.

65. Park C-K, Park S-H, Lee S-H, Kim C-Y, Kim D-W, Paek SH, Kim DG, Heo DS, Kim IH, Jung H-W: **Methylation status of the MGMT gene promoter fails to predict the clinical outcome of glioblastoma patients treated with ACNU plus cisplatin.** *Neuropathology* 2009, **29:**443-449.

66. Parrella P, la Torre A, Copetti M, Valori VM, Barbano R, Notarangelo A, Bisceglia M, Gallo AP, Balsamo T, Poeta ML, Carella M, Catapano D, Parisi S, Dallapiccola B, Maiello E, D'Angelo V, Fazio VM: **High specificity of quantitative methylation-specific PCR analysis for MGMT promoter hypermethylation detection in gliomas.** *J Biomed Biotechnol* 2009, **2009:**531692.

67. Hoque MO, Begum S, Topaloglu O, Chatterjee A, Rosenbaum E, Van Criekinge W, Westra WH, Schoenberg M, Zahurak M, Goodman SN, Sidransky D: **Quantitation of promoter methylation of multiple genes in urine DNA and bladder cancer detection.** *J Natl Cancer Inst* 2006, **98:**996-1004.

68. Paz MF, Yaya-Tur R, Rojas-Marcos I, Reynes G, Pollan M, Aguirre-Cruz L, Garcia-Lopez JL, Piquer J, Safont MJ, Balana C, Sanchez-Cespedes M, Garcia-Villanueva M, Arribas L, Esteller M: **CpG island hypermethylation of the DNA repair enzyme methyltransferase predicts response to temozolomide in primary gliomas.** *Clin Cancer Res* 2004, **10:**4933-4938.

69. Piccirilli M, Bistazzoni S, Gagliardi FM, Landi A, Santoro A, Giangaspero F, Salvati M: **Treatment of glioblastoma multiforme in elderly patients. Clinico-therapeutic remarks in 22 patients older than 80 years.** *Tumori* 2006, **92:**98-103.

70. Pirtoli L, Cevenini G, Tini P, Vannini M, Oliveri G, Marsili S, Mourmouras V, Rubino G, Miracco C: **The prognostic role of Beclin 1 protein expression in high-grade gliomas.** *Autophagy* 2009, **5:**930-936.

71. Prados MD, Chang SM, Butowski N, DeBoer R, Parvataneni R, Carliner H, Kabuubi P, Ayers-Ringler J, Rabbitt J, Page M, Fedoroff A, Sneed PK, Berger MS, McDermott MW, Parsa AT, Vandenberg S, James CD, Lamborn KR, Stokoe D, Haas-Kogan DA: **Phase II study of erlotinib plus temozolomide during and after radiation therapy in patients with newly diagnosed glioblastoma multiforme or gliosarcoma.** *J Clin Oncol* 2009, **27:**579-584.

72. Ramirez JL, Taron M, Balaña C, Sarries C, Mendez P, de Aguirre I, Nunez L, Roig B, Queralt C, Botia M, Rosell R: **Serum DNA as a tool for cancer patient management.** *Rocz Akad Med Bialymst* 2003, **48:**34-41.

73. Rivera AL, Pelloski CE, Gilbert MR, Colman H, De La Cruz C, Sulman EP, Bekele BN, Aldape KD: **MGMT promoter methylation is predictive of response to radiotherapy and prognostic in the absence of adjuvant alkylating chemotherapy for glioblastoma.** *Neuro Oncol* 2010, **12:**116-121.

74. Rodriguez FJ, Thibodeau SN, Jenkins RB, Schowalter KV, Caron BL, O'Neill B P, James CD, Passe S, Slezak J, Giannini C: **MGMT immunohistochemical expression and promoter methylation in human glioblastoma.** *Appl Immunohistochem Mol Morphol* 2008, **16:**59-65.

75. Sadones J, Michotte A, Veld P, Chaskis C, Sciot R, Menten J, Joossens EJ, Strauven T, D'Hondt LA, Sartenaer D, Califice SF, Bierau K, Svensson C, De Greve J, Neyns B: **MGMT promoter hypermethylation correlates with a survival benefit from temozolomide in patients with recurrent anaplastic astrocytoma but not glioblastoma.** *Eur J Cancer* 2009, **45:**146-153.

76. Schaich M, Kestel L, Pfirrmann M, Robel K, Illmer T, Kramer M, Dill C, Ehninger G, Schackert G, Krex D: **A MDR1 (ABCB1) gene single nucleotide polymorphism predicts outcome of temozolomide treatment in glioblastoma patients.** *Ann Oncol* 2009, **20:**175-181.

77. Shamsara J, Sharif S, Afsharnezhad S, Lotfi M, Raziee HR, Ghaffarzadegan K, Moradi A, Rahighi S, Behravan J: **Association between MGMT promoter hypermethylation and p53 mutation in glioblastoma.** *Cancer Invest* 2009, **27:**825-829.

78. Sijben AE, McIntyre JB, Roldán GB, Easaw JC, Yan E, Forsyth PA, Parney IF, Magliocco AM, Bernsen H, Cairncross JG: **Toxicity from chemoradiotherapy in older patients with glioblastoma multiforme.** *J Neurooncol* 2008, **89:**97-103.

79. Slaby O, Lakomy R, Fadrus P, Hrstka R, Kren L, Lzicarova E, Smrcka M, Svoboda M, Dolezalova H, Novakova J, Valik D, Vyzula R, Michalek J: **MicroRNA-181 family predicts response to concomitant chemoradiotherapy with temozolomide in glioblastoma patients.** *Neoplasma* 2010, **57:**264-269.

80. Smith KA, Ashby LS, Gonzalez LF, Brachman DG, Thomas T, Coons SW, Battaglia M, Scheck A: **Prospective trial of gross-total resection with Gliadel wafers followed by early postoperative Gamma Knife radiosurgery and conformal fractionated radiotherapy as the initial treatment for patients with radiographically suspected, newly diagnosed glioblastoma multiforme.** *J Neurosurg* 2008, **109:**106-117.

81. Sonoda Y, Kumabe T, Watanabe M, Nakazato Y, Inoue T, Kanamori M, Tominaga T: **Long-term survivors of glioblastoma: clinical features and molecular analysis.** *Acta Neurochir (Wien)* 2009, **151:**1349-1358.

82. Sonoda Y, Yokosawa M, Saito R, Kanamori M, Yamashita Y, Kumabe T, Watanabe M, Tominaga T: **O(6)-Methylguanine DNA methyltransferase determined by promoter hypermethylation and immunohistochemical expression is correlated with progression-free survival in patients with glioblastoma.** *Int J Clin Oncol* 2010, **15:**352-358.

83. Spiegl-Kreinecker S, Pirker C, Filipits M, Lotsch D, Buchroithner J, Pichler J, Silye R, Weis S, Micksche M, Fischer J, Berger W: **O6-Methylguanine DNA methyltransferase protein expression in tumor cells predicts outcome of temozolomide therapy in glioblastoma patients.** *Neuro Oncol* 2010, **12:**28-36.

84. Stupp R, Hegi ME, Neyns B, Goldbrunner R, Schlegel U, Clement PM, Grabenbauer GG, Ochsenbein AF, Simon M, Dietrich PY, Pietsch T, Hicking C, Tonn JC, Diserens AC, Pica A, Hermisson M, Krueger S, Picard M, Weller M: **Phase I/IIa study of cilengitide and temozolomide with concomitant radiotherapy followed by cilengitide and temozolomide maintenance therapy in patients with newly diagnosed glioblastoma.** *J Clin Oncol* 2010, **28:**2712-2718.

85. van den Bent MJ, Dubbink HJ, Sanson M, van der Lee-Haarloo CR, Hegi M, Jeuken JW, Ibdaih A, Brandes AA, Taphoorn MJ, Frenay M, Lacombe D, Gorlia T, Dinjens WN, Kros JM: **MGMT promoter methylation is prognostic but not predictive for outcome to adjuvant PCV chemotherapy in anaplastic oligodendroglial tumors: a report from EORTC Brain Tumor Group Study 26951.** *J Clin Oncol* 2009, **27:**5881-5886.

86. Vogelbaum MA, Berkey B, Peereboom D, Macdonald D, Giannini C, Suh JH, Jenkins R, Herman J, Brown P, Blumenthal DT, Biggs C, Schultz C, Mehta M: **Phase II trial of preirradiation and concurrent temozolomide in patients with newly diagnosed anaplastic oligodendrogliomas and mixed anaplastic oligoastrocytomas: RTOG BR0131.** *Neuro Oncol* 2009, **11:**167-175.

87. Watanabe T, Katayama Y, Komine C, Yoshino A, Ogino A, Ohta T, Fukushima T: **O6-methylguanine-DNA methyltransferase methylation and TP53 mutation in malignant astrocytomas and their relationships with clinical course.** *Int J Cancer* 2005, **113:**581-587.

88. Watanabe T, Katayama Y, Ogino A, Ohta T, Yoshino A, Fukushima T: **Preliminary individualized chemotherapy for malignant astrocytomas based on O6-methylguanine-deoxyribonucleic acid methyltransferase methylation analysis.** *Neurol Med Chir (Tokyo)* 2006, **46:**387-393.

89. Weiler M, Hartmann C, Wiewrodt D, Herrlinger U, Gorlia T, Bahr O, Meyermann R, Bamberg M, Tatagiba M, von Deimling A, Weller M, Wick W: **Chemoradiotherapy of newly diagnosed glioblastoma with intensified temozolomide.** *Int J Radiat Oncol Biol Phys* 2010, **77:**670-676.

90. Weller M, Felsberg J, Hartmann C, Berger H, Steinbach JP, Schramm J, Westphal M, Schackert G, Simon M, Tonn JC, Heese O, Krex D, Nikkhah G, Pietsch T, Wiestler O, Reifenberger G, von Deimling A, Loeffler M: **Molecular predictors of progression-free and overall survival in patients with newly diagnosed glioblastoma: a prospective translational study of the German Glioma Network.** *J Clin Oncol* 2009, **27:**5743-5750.

91. Wemmert S, Bettscheider M, Alt S, Ketter R, Kammers K, Feiden W, Steudel WI, Rahnenfuhrer J, Urbschat S: **p15 promoter methylation - a novel prognostic marker in glioblastoma patients.** *Int J Oncol* 2009, **34:**1743-1748.

92. Wick W, Hartmann C, Engel C, Stoffels M, Felsberg J, Stockhammer F, Sabel MC, Koeppen S, Ketter R, Meyermann R, Rapp M, Meisner C, Kortmann RD, Pietsch T, Wiestler OD, Ernemann U, Bamberg M, Reifenberger G, von Deimling A, Weller M: **NOA-04 randomized phase III trial of sequential radiochemotherapy of anaplastic glioma with procarbazine, lomustine, and vincristine or temozolomide.** *J Clin Oncol* 2009, **27:**5874-5880.

93. Yachi K, Watanabe T, Ohta T, Fukushima T, Yoshino A, Ogino A, Katayama Y, Nagase H: **Relevance of MSP assay for the detection of MGMT promoter hypermethylation in glioblastomas.** *Int J Oncol* 2008, **33:**469-475.

94. Yang S-H, Kim YH, Kim JW, Park C-K, Park S-H, Jung H-W: **Methylation Status of the O6-Methylguanine-Deoxyribonucleic Acid Methyltransferase Gene Promoter in World Health Organization Grade III Gliomas.** *J Korean Neurosurg Soc* 2009, **46:**385-388.

95. Zawlik I, Vaccarella S, Kita D, Mittelbronn M, Franceschi S, Ohgaki H: **Promoter methylation and polymorphisms of the MGMT gene in glioblastomas: a population-based study.** *Neuroepidemiology* 2009, **32:**21-29.

96. Palmisano WA, Divine KK, Saccomanno G, Gilliland FD, Baylin SB, Herman JG, Belinsky SA: **Predicting lung cancer by detecting aberrant promoter methylation in sputum.** *Cancer Res* 2000, **60:**5954-5958.

97. van Engeland M, Weijenberg MP, Roemen GMJM, Brink M, de Bruine AP, Goldbohm RA, van den Brandt PA, Baylin SB, de Goeij AF, Herman JG: **Effects of dietary folate and alcohol intake on promoter methylation in sporadic colorectal cancer: the Netherlands cohort study on diet and cancer.** *Cancer Res* 2003, **63:**3133-3137.

98. Alonso ME, Bello MJ, Gonzalez-Gomez P, Arjona D, Lomas J, de Campos JM, Isla A, Sarasa JL, Rey JA: **Aberrant promoter methylation of multiple genes in oligodendrogliomas and ependymomas.** *Cancer Genet Cytogenet* 2003, **144:**134-142.

99. Dong S-M, Pang JS-S, Poon W-S, Hu J, To K-F, Chang AR, Ng H-K: **Concurrent hypermethylation of multiple genes is associated with grade of oligodendroglial tumors.** *J Neuropathol Exp Neurol* 2001, **60:**808-816.

100. Everhard S, Kaloshi G, Crinière E, Benouaich-Amiel A, Lejeune J, Marie Y, Sanson M, Kujas M, Mokhtari K, Hoang-Xuan K, Delattre JY, Thillet J: **MGMT methylation: a marker of response to temozolomide in low-grade gliomas.** *Ann Neurol* 2006, **60:**740-743.

101. Houillier C, Wang X, Kaloshi G, Mokhtari K, Guillevin R, Laffaire J, Paris S, Boisselier B, Idbaih A, Laigle-Donadey F, Hoang-Xuan K, Sanson M, Delattre JY: **IDH1 or IDH2 mutations predict longer survival and response to temozolomide in low-grade gliomas.** *Neurology* 2010, **75:**1560-1566.

102. Kesari S, Schiff D, Drappatz J, LaFrankie D, Doherty L, Macklin EA, Muzikansky A, Santagata S, Ligon KL, Norden AD, Ciampa A, Bradshaw J, Levy B, Radakovic G, Ramakrishna N, Black PM, Wen PY: **Phase II study of protracted daily temozolomide for low-grade gliomas in adults.** *Clin Cancer Res* 2009, **15:**330-337.

103. Komine C, Watanabe T, Katayama Y, Yoshino A, Yokoyama T, Fukushima T: **Promoter hypermethylation of the DNA repair gene O6-methylguanine-DNA methyltransferase is an independent predictor of shortened progression free survival in patients with low-grade diffuse astrocytomas.** *Brain Pathol* 2003, **13:**176-184.

104. Kuo L-T, Kuo K-T, Lee M-J, Wei C-C, Scaravilli F, Tsai J-C, Tseng H-M, Kuo M-F, Tu Y-K: **Correlation among pathology, genetic and epigenetic profiles, and clinical outcome in oligodendroglial tumors.** *Int J Cancer* 2009, **124:**2872-2879.

105. Tosoni A, Franceschi E, Ermani M, Bertorelle R, Bonaldi L, Blatt V, Brandes AA: **Temozolomide three weeks on and one week off as first line therapy for patients with recurrent or progressive low grade gliomas.** *J Neurooncol* 2008, **89:**179-185.

106. Watanabe T, Nakamura M, Kros JM, Burkhard C, Yonekawa Y, Kleihues P, Ohgaki H: **Phenotype versus genotype correlation in oligodendrogliomas and low-grade diffuse astrocytomas.** *Acta Neuropathol* 2002, **103:**267-275.
